# Resolving the origins of invertebrate colonists in the Yangtze River Estuary with molecular markers: Implications for ecological connectivity

**DOI:** 10.1002/ece3.8095

**Published:** 2021-09-16

**Authors:** Yu‐Qiang Li, Meng‐Yu Li, Teng‐Fei Xing, Jin‐Xian Liu

**Affiliations:** ^1^ CAS Key Laboratory of Marine Ecology and Environmental Sciences Institute of Oceanology Chinese Academy of Sciences Qingdao China; ^2^ Laboratory for Marine Ecology and Environmental Science Qingdao National Laboratory for Marine Science and Technology Qingdao China; ^3^ Center for Ocean Mega‐Science Chinese Academy of Sciences Qingdao China; ^4^ University of Chinese Academy of Sciences Beijing China

**Keywords:** coastal current, *Littorina brevicula*, marine connectivity, Yangtze River Estuary, Yellow Sea

## Abstract

Understanding connectivity over different spatial and temporal scales is fundamental for managing of ecological systems. However, controversy exists for wintertime ecological connectivity between the Yangtze River Estuary (YRE) and inner southwestern Yellow Sea. Here, we investigated ecological connectivity between the YRE and inner southwestern Yellow Sea in wintertime by precisely pinpointing the source of the newly colonized populations of a winter‐spawning rocky intertidal invertebrate, *Littorina brevicula* (Philippi, 1844), on artificial structures along the coast of the Yangtze River Delta (YRD) using mitochondrial ND6 sequences and microsatellite data. Clear phylogeographic and genetic differentiation were detected between natural rocky populations south and north of the YRE, which resulted from the lack of hard substrate for rocky invertebrates in the large YRD coast. For the newly colonized populations on the coast of YRD, most individuals (98%) to the south of ~33.5°N were from natural rocky populations to the south of the YRE and most of those (94%) to the north of ~33.5°N were from the northern natural rocky populations, which demonstrated strong ecological connectivity between the inner southwestern Yellow Sea and the YRE in winter time. We presented the first genetic evidence that demonstrated a northward wintertime coastal current in the inner southwestern Yellow Sea, and precisely illustrated the boundary of the coastal current recently proposed by numerical experiment. These results indicated that the YRE serves as an important source of materials and energy for the inner southwestern Yellow Sea in winter, which can be crucial for the function of the Yellow Sea ecosystem.

## Graphical abstract

The study addressed a consequential question regarding the ecological connectivity between the Yangtze River Estuary and inner southwestern Yellow Sea. For the first time, we provided clear genetic evidence for strong ecological connectivity between the southwestern Yellow Sea and Yangtze River Estuary in winter season. The strong wintertime ecological connectivity indicated that the Yangtze River Estuary serves as an important source of materials and energy for the inner southwestern Yellow Sea in winter, which can be crucial for the function of the Yellow Sea ecosystem.

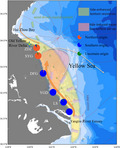

## 1 INTRODUCTION

Ecological connectivity is the movement of organisms, nutrients, and materials between populations, communities, ecosystems, or habitats, profoundly influencing the structure, functions, and dynamics of ecosystems (Cowen & Sponaugle, 2009; Taylor et al., 1993). Understanding connectivity over different spatial and temporal scales is fundamental for conserving and managing the diversity of biological systems (Jolly et al., 2009). Connectivity is also a fundamental ecological process in marine ecosystems, which influences gene flow, population dynamics, species interactions, patterns of distributions, and, ultimately, the functioning of systems (Balbar & Metaxas, 2019; Jeltsch et al., 2013). Knowledge on genetic structure of species is important to understand ecological connectivity and to define the spatiotemporal scales over which conservation management plans should be designed and implemented (Gandra et al., 2020). For benthic marine invertebrate with external fertilization and dispersive larvae, larval dispersal plays a fundamental role in population dynamics (Cowen & Sponaugle, 2009). Larval transport is a complex biological–physical process controlled by multiple factors include hydrodynamic processes caused by tide and wind‐induced currents, long‐term patterns of ocean currents, reproductive season, duration of the larval phase, and finally behavior and physiological plasticity of larvae (Pineda et al., 2018; Sponaugle et al., 2002).

The large shallow productive Yellow Sea is one of the 64 Large Marine Ecosystems (LMEs) in the world, which covers an area of 380,000 km^2^ with a mean depth of 44 m (Duda & Sherman, 2002; Tang, 2014; Tang et al., 2016). The Yellow Sea is one of the most heavily exploited LMEs and faces many challenges, including unsustainable fishery practices, harmful algal and jellyfish blooms, pollution, habitat modification, and other challenges (Li et al., 2015; Liu et al., 2017; Tang et al., 2006, 2016; Wang et al., 2015). The complex dynamic circulation system of the Yellow Sea has resulted in a highly complex and variable physical eco‐environment (Chen, 2009; Fu et al., 2009; Lie & Cho, 2016; Wei et al., 2016, 2018). The Subei Shoal in the southwestern Yellow Sea is a unique intertidal mudflat zone with an area around 22,740 km^2^ (Li, 2011). It extends over 200 km from the Sheyang River estuary to the Yangtze River Estuary (YRE) and 90 km from the shoreline to open sea. Since 2007, the world's largest trans‐regional green tide bloom, caused by *Ulva prolifera*, has been observed off the coast of the southern Yellow Sea in summer (Liu et al., 2013; Zhou et al., 2015). The green tide in Subei Shoal and the southern Yellow Sea may begin as green algal propagules (Zhou et al., 2015). Hydrographic factors may influence biogenic element regimes strongly in the Yellow Sea, especially the possible nutrient input of the Yangtze River plume into the Subei shoal (Li et al., 2015; Wu et al., 2018). However, controversy exists for the coast current in the inner southwestern Yellow Sea in winter. For decades, a southward coastal current, the Yellow Sea Coastal Current, was considered to exist in the southwestern Yellow Sea in wintertime Eastern Asian Monsoon (Guan, 1984, 1994; Ichikawa & Beardsley, 2002), which was widely accepted by marine ecologist working in this area (Guo et al., 2020; Ni et al., 2017; Wang et al., 2020; Zhou, 2010). However, recent observed data on two nearby stations in the Subei coast water demonstrated that subtidal transport in the inner southwestern Yellow Sea is also northward in wintertime, which is opposite to the downwelling‐favorable winter monsoon (Wu et al., 2018). Further numerical experiment illustrated that this northward coastal current exists from the YRE all the way along the Subei Coast until reaching ∼33.5°*N* (Wu et al., 2018). Transport of materials and energy from areas of high to low availability influences the types and numbers of organisms the recipient environment can support, as well as their biological interactions and, more broadly, the functioning of the ecosystem (Polis et al., 1997). To the south of the Yellow Sea, the YRE is a major source of terrigenous materials, and the Yangtze River discharges 924 billion m^3^ of freshwater annually (∼2.4% of the global total) as well as huge amount of nutrients and sediments (Gao & Wang, 2008). Because the occurrence of the macroalgal bloom was related to high nutrient levels (Li et al., 2007; Liu, 2013), investigating the ecological connectivity between the YRE and inner southwestern Yellow Sea is essential for a comprehensive understanding of the mechanisms underlying the major processes in this area.

**FIGURE 1:**
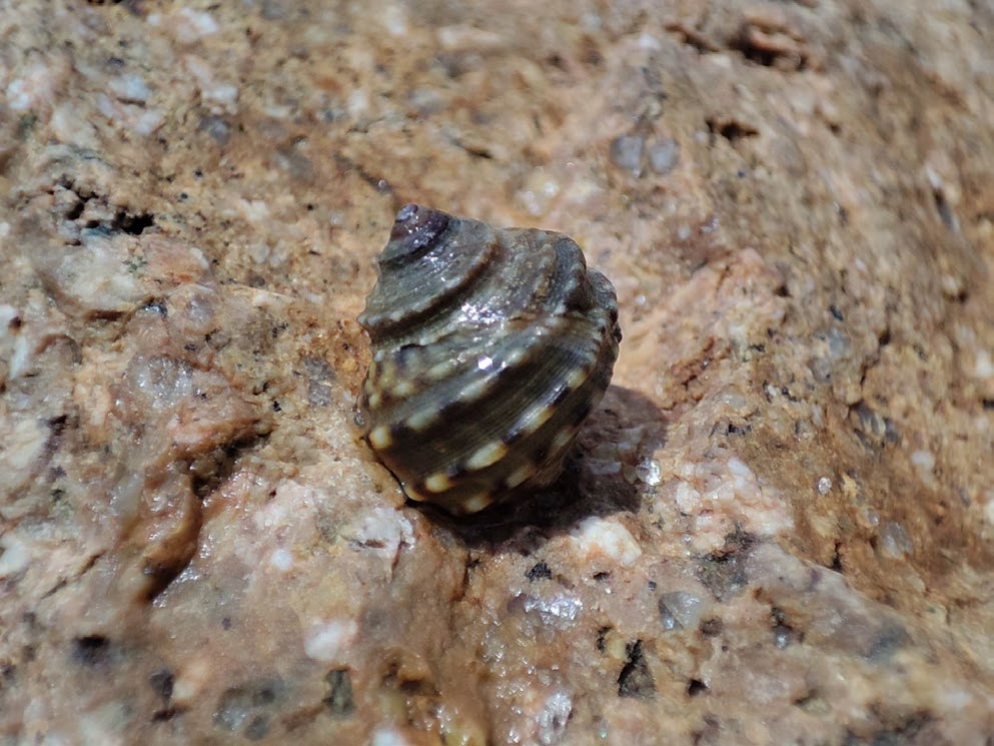
The *Littorina brevicula* in its natural rocky intertidal habitats of Qingdao, China

*Littorina brevicula* (Philippi, 1844) (Figure 1) is a common periwinkle snail distributed in the rocky littoral fringe of the temperate coast of the Northwest Pacific, ranging from Hong Kong to Peter the Great Bay along the continental coast of Asia (Okutani, 2000; Reid, 1996). The spawning season of *L. brevicula* is from January to April when seawater temperature is low (~12℃) (Kojima, 1957; Son & Hong, 1998). The egg capsule is pelagic and veliger hatch about 7 days after spawning (Reid, 1996; Son & Hong, 1998). The duration of the planktonic egg and larval period is several weeks (Golikov, 1976). Within the distribution range of *L. brevicula*, the Yangtze River Delta (YRD) is an alluvial plain created by activities of the Yangtze River, Old Yellow River, and other rivers and is characterized by muddy coastline that is inappropriate for settlement of rocky intertidal species from the Yangtze River mouth (c. 30°N) to Lianyungang, Jiangsu (c. 34.5°N) (Chen et al., 1999; Dong et al., 2016). For marine organisms with a life history phase that is dependent on hard substrate for larval settlement, the muddy coastline of the YRD can be a natural barrier to dispersal (Ni et al., 2017; Wang et al., 2020). However, with human population growth and land reclamation, the coast of the YRD is increasingly dominated by artificial structures defending against rising and stormier seas (e.g., seawalls, groynes, and breakwaters) (Dong et al., 2016; Huang et al., 2015). These artificial structures along coastal areas of the YRD provide suitable habitats for the settlement of intertidal rocky‐shore species (Huang et al., 2015). Meanwhile, it can also have large‐scale impacts through their alteration of ecological connectivity by the movement of organisms, materials, and energy between habitat units within seascapes (Adams et al., 2014). Indeed, newly colonized populations of *L. brevicula* have been found on the artificial rocky shores throughout the coast of the YRD (Dong et al., 2016). Considering its distribution on natural rocky shores both south and north to the YRD and wintertime spawning, *L. brevicula* therefore constitutes an ideal biological model to elucidate connectivity between the inner southwestern Yellow Sea and the YRE in winter monsoon season by checking the source of the newly colonized populations on the coast of YRD.

The aim of the present work was to clarify the ecological connectivity between the inner southwestern Yellow Sea and the YRE in winter monsoon season by precisely pinpointing the source of the newly colonized populations of *L. brevicula* on the artificial structures along coastal areas of the YRD. By using complete mitochondrial ND6 gene sequences and microsatellite markers, we inferred the source of the newly colonized populations by investigating spatial genetic structure among the newly colonized populations on coasts of YRD and natural rocky intertidal populations south and north to the YRD. For the first time, our results clearly demonstrated that most of the individuals of the newly colonized populations to the south of ~33.5°N were from natural populations to south of the YRE and those to the north of 33.5°N were from natural populations to north of the YRD. These results suggested a strong ecological connectivity between the inner southwestern Yellow Sea and the YRE in winter, which can be crucial for the function of the Yellow Sea ecosystem and have general interest to marine ecologist working in this area.

## 2 MATERIAL AND METHODS

### 2.1 Ethics statement

The field studies did not involve any endangered or protected species. *L*. *brevicula* is not protected by Chinese law. Procedures were performed in accordance with the Regulations for the Administration of Affairs Concerning Experimental Animals of the Institute of Oceanology, Chinese Academy of Sciences.

### 2.2 Sample collection

**FIGURE 2:**
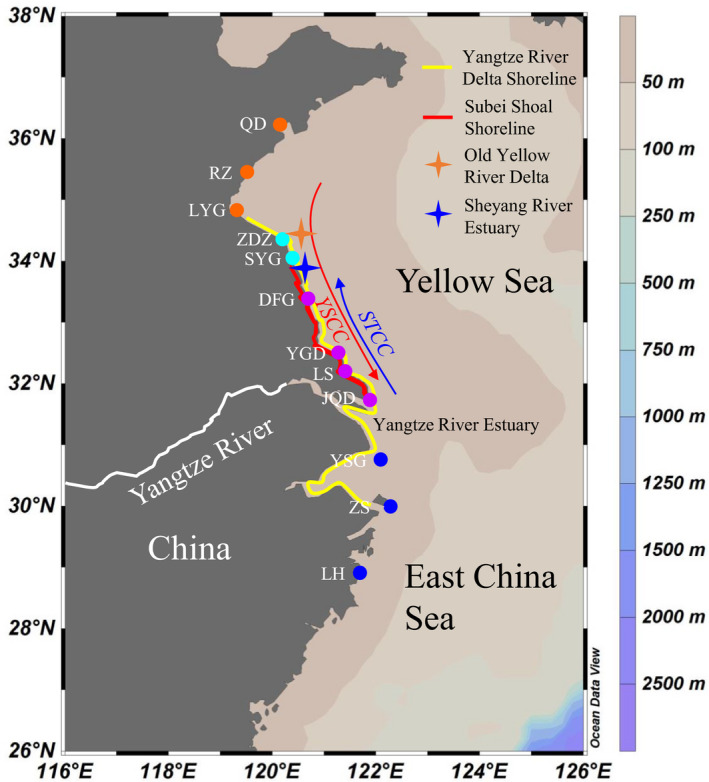
Map of the sampling location for twelve populations of *L. brevicula* and maps of the coastal currents along the southwestern Yellow Sea in winter. The Yellow Sea Coastal Current flows from north to south, and the Subei tide‐induced coastal current flows from south to north. The 4‐ray stars represent the Old Yellow Sea Delta and the Sheyang River Estuary, respectively. The colors of the sampling locations correspond to those illustrated in Figure 3. Figure was generated by Ocean Data View 5.0.0 (Schlitzer, Reiner., Ocean Data View, http://odv.awi.de, 2018)

A total of 290 individuals of *L. brevicula* were collected from 12 geographic localities covering the YRD coast artificial structures and nearby natural rocky intertidal areas from 2018 to 2019 (Figure 2 and Table 1). The samples were divided into three groups according to their geographic origins. Three samples collected from natural rocky intertidal shore to the north of the YRD (Qingdao, Rizhao, and Lianyungang) were defined as northern natural populations. Six samples collected on artificial structures along the coastlines of the YRD (Zhendongzha, Shuangyanggang, Dafenggang, Yangguangdao, Lvsi, and Qidong) were grouped as newly colonized populations. Three samples collected from natural rocky intertidal areas to the south of the YRE (Yangshangang, Zhoushan, and Linhai) were defined as southern natural populations. Foot muscle tissues of the specimens were fixed and preserved in 95% ethanol.

**TABLE 1:** Sample sites and summary statistics^a^ of the mitochondrial ND6 genetic diversity for *Littorina brevicula* on the coast of Yangtze River Delta

Region/Source	Sample site	Abbreviation	Coordinates	Collection date	Sample size	No. of variable sites	No. of haplotypes	*h*	*π*
Northern Natural populations	Qingdao	QD	36°03'*N*; 120°20'E	2018.03.06	24	3	4	0.24	0.0005
	Rizhao	RZ	35°30'*N*; 119°38'E	2018.04.16	24	2	3	0.16	0.0003
	Lianyungang	LYG	34°46'*N*; 119°20'E	2018.07.06	24	4	5	0.31	0.0007
Newly colonized populations on artificial substrum	Zhendongzha	ZDZ	34°07'*N*; 120°11'E	2018.07.06	24	10	7	0.45	0.0018
	Shuangyanggang	SYG	34°00'*N*; 120°24'E	2018.07.30	24	10	7	0.45	0.0016
	Dafenggang	DFG	33°12'*N*; 120°49'E	2018.07.07	24	8	8	0.66	0.0024
	Yangguangdao	YGD	32°26'*N*; 121°20'E	2018.07.07	24	14	10	0.86	0.0044
	Lvsi	LS	32°04'*N*; 121°20'E	2018.04.17	25	8	9	0.86	0.0041
	Qidong	JQD	31°26'*N*; 121°33'E	2019.04.09	25	13	12	0.77	0.0031
Southern Natural populations	Yangshanggang	YSG	30°38'*N*; 122°03'E	2018.8.30	24	6	6	0.77	0.0034
	Zhoushan	ZS	29°32'*N*; 122°16'E	2018.04.18	24	9	9	0.70	0.0022
	Linhai	LH	28°50'*N*; 121°40'E	2018.04.29	24	13	12	0.83	0.0041

^a^ No. of variable sites: number of variable sites; No. of haplotypes: number of haplotypes; *h*: haplotype diversity; *π*: nucleotide diversity.

### 2.3 DNA extraction, mitochondrial DNA sequencing, microsatellite development, and genotyping

Total genomic DNA was extracted from a piece of foot muscle using a standard phenol chloroform method. The amplification of the complete mitochondrial NADH dehydrogenase subunit 6 (mt ND6) gene was performed by PCR in a 25‐μl reaction mixture containing template DNA (1 μl), ddH2O (10.5 μl), 2X PCR Mix (12.5 μl), and a pair of “*Littorina* specific” primers [Lbnd‐*F* (5’‐AGG TAC ATA TTC CTG CGC TCT GAA A‐3’) and Lbnd‐R (5’‐GTG TGC GCA TGA AAT GTA T‐3’)] (10 μM, respectively) (Kim et al., 2003). The thermal cycling profile included precycling denaturation at 95 ℃ for 5 min, followed by 35 cycles of denaturation at 95 ℃ for 30 s, annealing at 50 ℃ for 40 s, extension at 72 ℃ for 1 min, and a final extension at 72 ℃ for 10 min. The PCR products were checked by electrophoresis on 2% agarose. Then, PCR products were gel purified using gel extraction kit and cycle‐sequenced using the BigDye™ Terminator Cycle Sequencing Kit V. 3.1 on an ABI 3730xl DNA Analyzer (Applied Biosystems) at biotechnology company TingKe (Qingdao, China). All the samples were sequenced with both forward and reverse primers, which were the same as those for PCR amplification.

For the development of microsatellite loci, a de novo genomic library with insert size ~350bp was constructed following a standard Illumina protocol. Sequencing was performed to generate 150‐bp paired‐end reads on the HiSeq X Ten platform (Illumina) at Novogene Bioinformatics Technology (Tianjin, China). Then, de novo assembly was carried out using MEGAHIT using default settings (Li, Liu, et al., 2015). The scaffolds were then used for further microsatellite isolation and discovery. Microsatellite discovery and primer design were performed with QDD 3.1.2 (Meglécz et al., 2010) on a local Galaxy platform. Sequences with microsatellites (pure tandem repeats of di‐ to hexa‐nucleotide motif with at least ten uninterrupted repeats) and with at least 150 bases of flanking sequences on both sides were detected for primers design in Primer3 (Rozen & Skaletsky, 2000). Finally, a total of 12 newly developed polymorphic microsatellite loci (Table S1) were used for subsequent analysis. The amplification of the 12 microsatellite loci was performed by the PCR protocol described in Liu and Avise (2011). The thermal cycling parameters were the same as that previously described for mt ND6 except annealing temperatures (53–58℃ for different loci (Table S1)). PCR products with different fluorescent labels were pooled and electrophoresed on an ABI 3730xl automated sequencer (Applied Biosystems) at Sangon Biological Technology (Qingdao, China). Allele sizes were determined with the GS500‐ROX size standard using GeneMarker 2.2.0 (SoftGenetics, State College, USA).

### 2.4 Mitochondrial genetic variation and phylogeographic structure

The complete mt ND6 sequences were edited and aligned using the DNASTAR software (DNASTAR, Inc., Madison, WI, USA). Molecular diversity indices such as haplotype diversity (*h*), nucleotide diversity (π), number of polymorphic sites, haplotypes, transversions, and transitions were obtained using the program DnaSP 5 (Librado & Rozas, 2009). Genealogical relationships among mt ND6 haplotypes were investigated with the median‐joining network approach (Bandelt et al., 1999) as implemented in PopART (Leigh & Bryant, 2015) ( http://popart.otago.ac.nz).

### 2.5 Genetic diversity analysis of microsatellites

Microsatellite genotypes were scored using GeneMarker 2.2.0. Genetic diversity indices including number of alleles (*N*a), allelic richness (*AR*), and inbreeding coefficient (*F*_IS_) for each population were calculated using FSTAT 2.9.3 (Goudet, 2001). Measures of genetic diversity within each population including the observed heterozygosity (*H*_O_), expected heterozygosity (*H*_E_), and polymorphic information content (*PIC*) were calculated using Excel Microsatellite Toolkit (Park, 2001). Departure from Hardy–Weinberg equilibrium (HWE) and genotypic linkage disequilibrium (LD) was estimated by GENEPOP 4.0 (Rousset, 2008). Standard Bonferroni correction for multiple comparisons (α = 0.05) was applied to determine the significance of HWE and LD tests. The software Micro‐checker 2.2.3 (Oosterhout et al., 2004) was used to detect the presence of null alleles.

### 2.6 Population genetic structure

Pairwise *Φ*_ST_ for mt ND6 and *F*_ST_ for microsatellites among populations was calculated using ARLEQUIN 3.5, and significance was assessed by 10,000 bootstrap permutations (Excoffier & Lischer, 2010). Genetic relationships among populations were assessed by multidimensional scaling analyses (MDS) based on the mt DNA *Φ*_ST_ using the “vegan” (Dixon, 2003) packages in R 3.2.2 software.

Population genetic structure was then assessed through Bayesian clustering of microsatellite genotypes as implemented in STRUCTURE 2.3.4 (Pritchard et al., 2000). Ten independent runs were conducted for different numbers of genetic clusters (K = 1 to K = 12), each with a burn‐in period of 100,000 followed by 1,000,000 MCMC iterations, under an admixture model and correlated allele frequencies, and with no prior knowledge of sampling locations. To determine the most probable value of K, the Delta K method (Evanno et al., 2005) was used as implemented in Structure Selector (Li & Liu, 2018). The software CLUMPAK (Kopelman et al., 2015) was used to create a graphical representation of the STRUTURE outputs. Furthermore, to assess relationships among multilocus genotypes, we performed a discriminant analysis of principal components (DAPC) (Jombart et al., 2010) using the “adegenet” package (Jombart, 2008) in R 3.2.2 software, with the number of principle components set to 11 following alpha‐score indication. DAPC is a multivariate, model‐free approach designed to clustering based on prior population information (Jombart et al., 2010).

### 2.7 Assignment and exclusion tests

By using the multilocus microsatellite genotype data, the source of the 146 individuals in the six newly colonized populations was explored by the genetic assignment/exclusion tests conducted by the direct and simulation approaches implemented in GENECLASS 2.0 (Piry et al., 2004). The three northern natural populations were grouped as the northern reference population, and the three southern natural populations were grouped as southern reference population. Assignment tests were performed to assign individuals of the six newly colonized populations to the two reference populations. The reference population with the probability of assignment >95% was considered as the most likely source for the assigned genotype. Unlike the assignment approach, the exclusion method does not assume that the true population of origin has been sampled, and can exclude all reference populations as putative sources of introduced populations (Cornuet et al., 1999). Exclusion tests were performed using the resampling algorithm of Paetkau et al. (2004). All simulations were conducted with 100,000 simulated individuals and an alpha level of 0.01.

## 3 RESULTS

### 3.1 Summary statistics of molecular diversity

A 513‐bp sequence of the complete mtDNA ND6 gene was obtained for 290 individuals from 12 localities. Comparison of sequences revealed 54 distinct haplotypes defined by 53 polymorphic sites with 50 transitions and 4 transversions. Most of the haplotypes (45/83%) were singletons represented by a single individual. Of the remaining nine haplotypes, eight were shared among populations and one was found in two individuals but in one population (Table S2). For the natural rocky populations, the haplotype diversity (*h*) and nucleotide diversity (*π*) in the northern natural populations were much lower than in the southern natural populations, which was also consistent with results of the number of polymorphic sites and haplotypes. For the newly colonized populations, both *h* and *π* were also lower in the two northern populations than in the four populations on the coastline of the Subei Shoal (Table 1).

Out of 144 microsatellite locus–population combinations, 46 were significantly deviated from HWE after Bonferroni correction (Table S3). After Bonferroni correction, genotypic linkage disequilibrium was detected only between one pair of loci in Yangguangdao (Table S4). Four loci (Lbr10, Lbr15, Lbr41 and Lbr43) showed evidence of null alleles in most populations (9 ~ 12) according to Micro‐checker analysis (Table S5). Evidence of null alleles was detected in five other microsatellite loci in one (Lbr44) to seven populations (Lbr25). The distribution of null alleles among populations showed geographic patterns on some loci, some only detected in populations to the south of Dafenggang (Lbr25, Lbr32) and some only in the newly colonized populations (Lbr40), possibly reflected geographic differentiation or nonequilibrium populations. Null microsatellite alleles are found in most taxa but seem to be particularly common in populations with high effective population sizes including mollusks (Chiesa et al., 2016; Li et al., 2003; Rico et al., 2017). Furthermore, null alleles were likely to be encountered in populations that have diverged from the population from which the cloned allele state was drawn and the primers designed (Chapuis & Estoup, 2006). Indeed, the microsatellites in the present study were developed based on genomic sequences of an individual from the northern population Qingdao, which was consistent with the observation of null alleles only in the southern populations on some microsatellite loci (Lbr25, Lbr32, Lbr40). Simulation studies and empirical data sets showed that null alleles with frequencies between 5% and 8% should have only minor effects on classical estimates of population differentiation, although higher null allele frequencies may inflate *F*_ST_ and genetic distances (Chapuis & Estoup, 2006). Indeed, the *F*_ST_, STRUCTURE, and DAPC analysis generated similar results with or without the four loci that showed null alleles in most populations (Table S7, Figure S3, and Figure S4). Hence, we retained all loci for further population genetic analysis. The number of alleles per locus ranged from 9 (Lbr31) to 32 (Lbr15) with an average of 18.75. Average allele richness across microsatellite loci ranged from 8.20 in Qingdao to 10.49 in Lvsi. Average expected heterozygosity of each microsatellite across all samples ranged from 0.71 to 0.77, with a mean of 0.74 across all loci. Average observed heterozygosity of each microsatellite across all samples ranged from 0.46 to 0.64 with a mean of 0.53 across all loci. Detailed information for the 12 microsatellite loci in 12 populations is shown in Table S6.

### 3.2 Genealogy and geographic distribution of mt ND6 haplotypes

**FIGURE 3:**
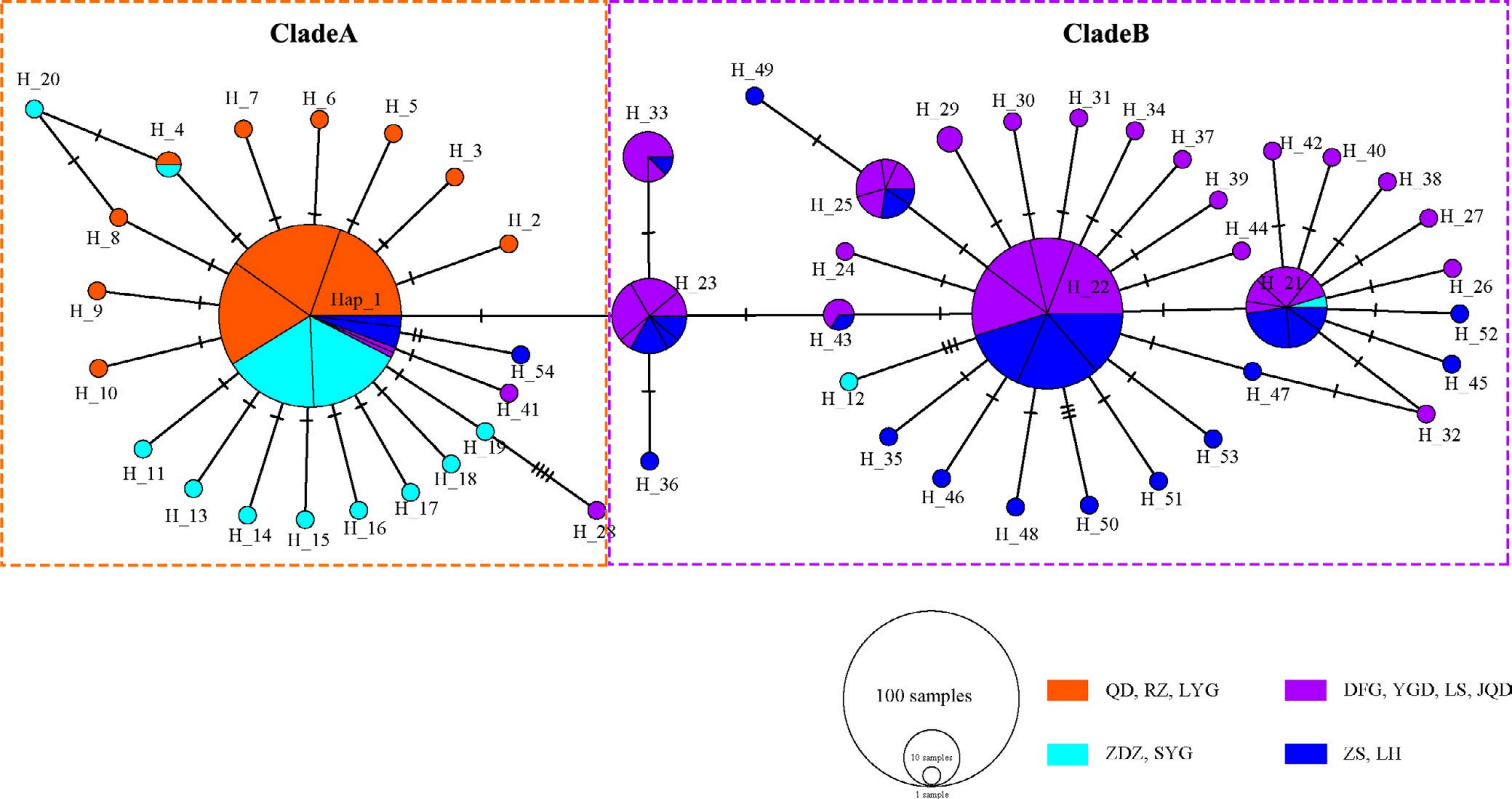
Median‐joining network for the 54 mt ND6 haplotypes of *L. brevicula* constructed in PopART. Haplotypes are represented by circles, the sizes of which are proportional to the number of individuals. Different colors represent geographic distribution. Mutational steps between haplotypes are indicated by hatch marks

The median‐joining network of the 54 haplotypes observed in the 290 individuals revealed a shallow topology with two clades (hereafter clade A and B) separated from each other by only a single mutational step. A total of 129 individuals with 22 haplotypes were detected in clade A, and 161 individuals with 32 haplotypes were found in clade B. Network of clade A was star‐like with a dominant haplotype H1 (107 individuals, ∼37%) in the center (Figure 3, Table S2). In clade B, a dominant haplotype H22 (72 individuals, ∼25%) forms the center of a star‐like network (Figure 3, Table S2). The two clades were heterogeneously distributed in the natural rocky intertidal populations. When considering the natural rocky intertidal populations only, haplotypes of clade B were only distributed in the three southern natural rocky populations. All haplotypes of individuals in the three northern natural populations were found in clade A. However, two clade A haplotypes (H1 and H54) were also found in seven individuals in the three southern natural population, six individuals including four from Yangshangang and two from Linhai with H1, and one individual from Linhai with H54 (Figure 3, Table S2). Considering the newly colonized populations on YRD, haplotypes observed in 94 of the 98 individuals (96%) in the four newly colonized populations on artificial structures along the coastlines of the Subei Shoal were distributed on clade B. The other four individuals were found with haplotypes in clade A, two individuals in Dafenggang and Yangguangdao with the most dominant haplotype H1, one individual in Yangguangdao with H28, and one from Qidong with H41. Haplotypes of 46 of the 48 individuals (96%) in the two newly colonized populations north of the Subei Shoal were on clade A. The other two individuals, each from Shuangyanggang and Zhendongzha, were found with haplotypes H12 and H21 in clade B.

### 3.3 Population genetic structure

The pairwise *Φ*_ST_ values of mt ND6 between the five populations north of 33.5°*N* (hereafter Northern Populations) and the seven populations south of 33.5°*N* (hereafter Southern Populations) were strong (*Φ*_ST_ = 0.426–0.572) and were all statistically significant after Bonferroni's correction (Table 2). However, there were no significant values of *Φ*_ST_ among pairs of the Northern Populations and pairs of the Southern Populations (*Φ*_ST_ = −0.027–0.052). For microsatellite data, the pairwise *F*_ST_ values between the Northern Populations and Southern Populations ranged from 0.069 to 0.123 and were all statistically significant after Bonferroni's correction (Table 3). Consistent with the mt DNA, none of the *F*_ST_ among pairs of the Northern Populations and pairs of the Southern Populations were statistically significant.

**TABLE 2:** Pairwise *Φ*_ST_ (below diagonal) and the corresponding *p*‐values (above diagonal) between populations of *Littorina brevicula* on the coast of Yangtze River Delta based on mtDNA data. All pairwise *Φ*_ST_ in bold are significant after FDR correction (*p*‐value < .0008)

	QD	RZ	LYG	ZDZ	SYG	DFG	YGD	LS	JQD	YSG	ZS	LH
QD	0.000	1.000	1.000	0.459	0.465	0.000	0.000	0.000	0.000	0.000	0.000	0.000
RZ	−0.016		0.663	0.249	0.248	0.000	0.000	0.000	0.000	0.000	0.000	0.000
LYG	−0.017	−0.005		0.733	0.725	0.000	0.000	0.000	0.000	0.000	0.000	0.000
ZDZ	−0.001	0.026	−0.010		1.000	0.000	0.000	0.000	0.000	0.000	0.000	0.000
SYG	0.004	0.026	−0.010	−0.019		0.000	0.000	0.000	0.000	0.000	0.000	0.000
DFG	**0.534**	**0.572**	**0.497**	**0.430**	**0.428**		0.072	0.056	0.421	0.273	1.000	0.618
YGD	**0.428**	**0.467**	**0.392**	**0.325**	**0.319**	0.041		0.181	0.185	0.596	0.119	0.473
LS	**0.476**	**0.514**	**0.440**	**0.373**	**0.371**	0.052	0.020		0.178	0.076	0.060	0.286
JQD	**0.467**	**0.505**	**0.431**	**0.364**	**0.362**	0.000	0.016	0.020		0.209	0.661	0.947
YSG	**0.410**	**0.450**	**0.373**	**0.306**	**0.299**	0.012	−0.010	0.042	0.015		0.279	0.742
ZS	**0.529**	**0.567**	**0.493**	**0.426**	**0.423**	−0.027	0.028	0.046	−0.010	0.010		0.656
LH	**0.426**	**0.465**	**0.389**	**0.322**	**0.319**	−0.009	−0.003	0.009	−0.019	−0.016	−0.010	

**TABLE 3:** Pairwise *F*_ST_ values (below diagonal) and the corresponding *p*‐values (above diagonal) between populations of *Littorina brevicula* on the coast of Yangtze River Delta based on 12 microsatellite loci. All pairwise *F*_ST_ values in bold are significant after FDR correction (*p* < .0008)

	QD	RZ	LYG	ZDZ	SYG	DFG	YGD	LS	JQD	YSG	ZS	LH
QD		0.318	0.067	0.124	0.028	0.000	0.000	0.000	0.000	0.000	0.000	0.000
RZ	0.006		0.058	0.215	0.509	0.000	0.000	0.000	0.000	0.000	0.000	0.000
LYG	0.011	0.012		0.352	0.039	0.000	0.000	0.000	0.000	0.000	0.000	0.000
ZDZ	0.010	0.008	0.006		0.208	0.000	0.000	0.000	0.000	0.000	0.000	0.000
SYG	0.014	0.004	0.013	0.008		0.000	0.000	0.000	0.000	0.000	0.000	0.000
DFG	**0.090**	**0.081**	**0.082**	**0.076**	**0.096**		0.008	0.190	0.001	0.088	0.310	0.120
YGD	**0.113**	**0.115**	**0.105**	**0.115**	**0.123**	0.019		0.005	0.040	0.134	0.105	0.103
LS	**0.084**	**0.072**	**0.069**	**0.074**	**0.077**	0.010	0.020		0.459	0.118	0.119	0.784
JQD	**0.097**	**0.086**	**0.082**	**0.088**	**0.095**	0.027	0.016	0.007		0.316	0.088	0.460
YSG	**0.103**	**0.098**	**0.095**	**0.100**	**0.111**	0.012	0.010	0.011	0.008		0.346	0.177
ZS	**0.101**	**0.097**	**0.092**	**0.092**	**0.106**	0.008	0.012	0.012	0.015	0.007		0.309
LH	**0.079**	**0.079**	**0.075**	**0.077**	**0.086**	0.012	0.012	0.003	0.007	0.010	0.009	

**FIGURE 4:**
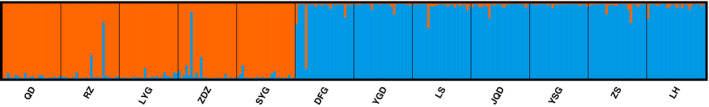
Population genetic structure analysis for microsatellite genotypes of *L. brevicula* implemented in STRUCTURE. Structure clustering results obtained at K = 2 is shown. Barplots showing posterior probabilities of individual genotypes (as bars) assigned to each population. The black lines separate sampling localities

**FIGURE 5:**
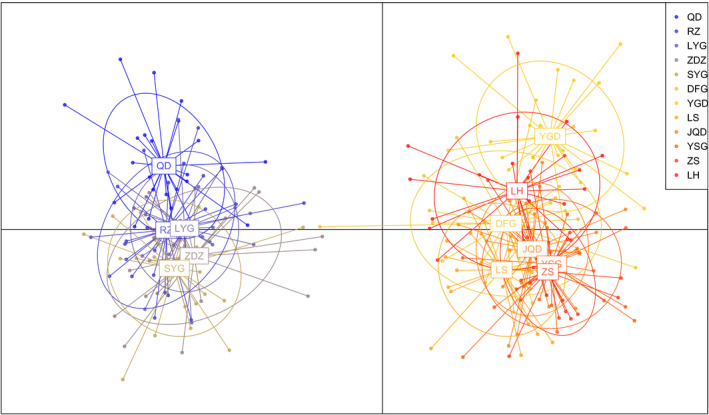
Scatter plots depicting the first two principal components of the discriminant analysis of principal components (DAPC) for assessment of 12 populations of *L. brevicula* based on microsatellite data. The scatter plot shows the clustering of individuals of *L. brevicula* into the groups represented by the inertia eclipses

The multidimensional scaling (MDS) plot of pairwise *Φ*_ST_ values for mt ND6 clearly identified two geographically restricted groups of populations, each representing the Northern Populations and Southern Populations, respectively (Figure S1). Consistent with the mt ND6 MDS analyses, the STRUCTURE analysis of microsatellite indicated highest support for two (K = 2 with the highest Delta K (Figure S2)) clusters among the populations, with the five Northern Populations forming one cluster and the rest seven Southern Populations grouping into another cluster (Figure 4). The DAPC analyses of microsatellites provided support for the genetic differentiation indicated by the MDS of mt ND6 and STRUCTURE analyses of microsatellites. DAPC clustering using the first two principal components, explaining 78.95% variance in total, showed that individuals from the Northern Populations were overlapped, and the ellipses of the Southern Populations were grouped together tightly (Figure 5).

### 3.4 Source of individuals in the newly colonized populations

**FIGURE 6:**
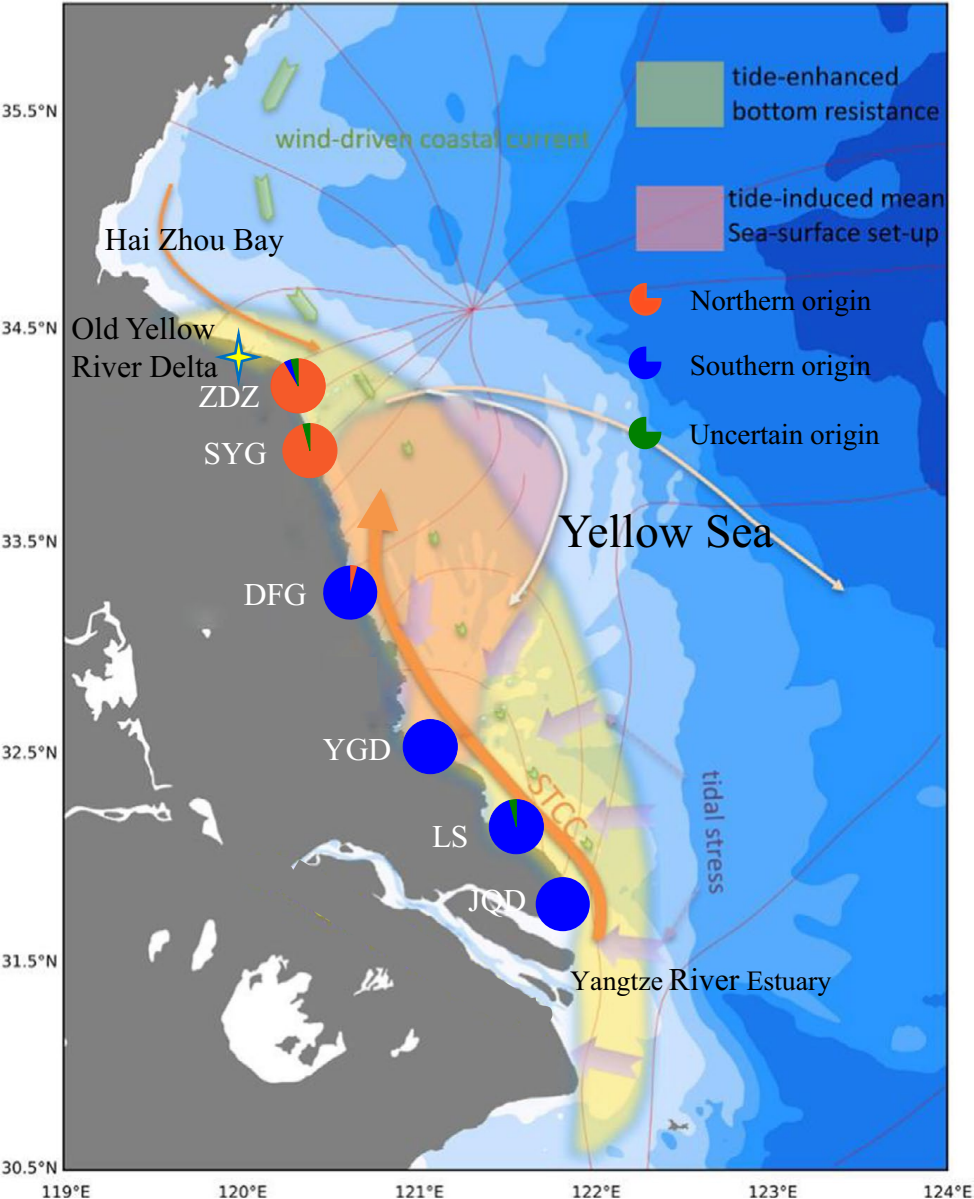
The source of individuals of the six newly colonized populations on the artificial structures along the coast of Yangtze River Delta inferred by the genetic assignment/exclusion tests based on microsatellite genotype data. The maps were used with permission from the author (Hui Wu)

Although simulation studies showed that bias in assignment tests caused by null alleles lead to a slight reduction in the power to correctly assigned individuals (2.4 percent units for GENECLASS‐based assignment tests), microsatellite loci affected by null alleles would not alter the overall outcome of assignment testing and could therefore be included in the analysis (Carlsson, 2008), so the assignment and exclusion test in the present study were carried out by using all the microsatellite loci. Most (45 or 94%) of the 48 individuals in the two newly colonized populations north of 33.5°N were assigned to the northern reference population with high assignment probability (> 95%). One individual (ZDZ06) with haplotype H12 in clade B was assigned to the southern reference population with a high assignment probability of 99%. The remaining two individuals (ZDZ04 and SYG16) with haplotype H1 in clade A were also assigned to the northern reference population, but with assignment probability of only 60% and 77%, respectively. Most (96 or 98%) of the 98 individuals in the four newly colonized populations on coastlines of the Subei Shoal were assigned to the southern reference population with high assignment probability (> 95%). One individual (DFG05) was assigned to the northern reference population with a high assignment probability of 98%. The other one (LS07) was also assigned to the southern reference population but with a low assignment probability of 69%. Results of the exclusion test were consistent with that of the assignment test. Exclusion probabilities of all the individuals with high assignment probability (> 95%) from their assigned reference population were much higher than those from the unassigned reference population, supporting the validity of the assignment test (Figure 6, Table S8). Results of the assignment and exclusion test without the four loci that showed null alleles in most populations were consistent with the results based on all the loci (Table S9).

## 4 DISCUSSIONS

Understanding of larval transport process is important for achieving an understanding of larval dispersal and connectivity (Cowen & Sponaugle, 2009; Pineda et al., 2018). In the present study, we clearly demonstrated strong ecological connectivity between the inner southwestern Yellow Sea and the YRE in winter monsoon season by precisely pinpointing the source of the newly colonized populations of *L. brevicula* on the artificial structures along coastal areas of the large YRD. Such knowledge has important implications for the management and conservation of the Yellow Sea ecosystem.

Knowledge on genetic structure is key to understand species connectivity patterns and to define the spatiotemporal scales over which conservation management plans should be designed and implemented (Gandra et al., 2020). However, the lack of genetic differentiation among coastal populations at regional scales usually hinders the understanding of the marine connectivity. Dong et al. (2016) investigated the phylogeographic pattern of *L. brevicula* and inferred the source of populations on the artificial structures on coastline of the YRD by using mitochondrial COI sequences. By analyses of population genetic structure, they concluded that most populations on the artificial structures showed genetic affinity with the natural rocky populations south of the YRE. However, one major haplotype found in 81% individuals was widely distributed and dominant in all the natural and newly colonized populations analyzed in their study, which makes the inference of origin of individuals incomplete by using low‐resolution mitochondrial COI sequences. In our study, the shallow but clear genetic/phylogeographic differentiation detected between the natural rocky populations of *L. brevicula* south and north of the YRE provided an excellent opportunity to explore the ecological connectivity between the inner southwestern Yellow Sea and the YRE. Considering the distinct geographic distribution of the two mitochondrial lineages and the clear genetic differentiation of microsatellite data, the lack of hard substrate in the large YRD for the rocky intertidal invertebrates, rather than the Yangtze River outflow, should present a larval dispersal barrier for *L. brevicula* and lead to the genetic divergence between the two observed genetic groups. Considering the shallow divergence between the two mitochondrial clades (separated by only a single mitochondrial mutational step), their distinct spatial distribution in natural rocky populations south and north of the YRE, and the cyclic nature of the Pleistocene glaciations, the phylogeographic divergence of the two mitochondrial lineages could have occurred after populations of *L. brevicula* recolonized the natural rocky habitats from a common glacial refugia after the last glacial maximum when the sea level rose. Indeed, the great drainages of the world produce genetic partitions in coastal marine organisms, such as the Yangtze River outflow (Ni et al., 2017) and the Amazon River outflow (Floeter et al., 2008).

Connectivity influences ecological systems at levels of organization ranging from genes to ecosystems (Sheaves, 2009). Both mitochondrial ND6 and microsatellites clearly demonstrated that most (≧94%) of the individuals in the two newly colonized populations north of 33.5°N were from the northern natural populations, and most (≧98%) of the individuals in the four newly colonized populations south of 33.5°N were from the southern natural populations. Also, results of all the population genetic analyses strongly supported two geographically restricted and genetically differentiated groups representing the populations north and south of 33.5°N, respectively. These results set 33.5°N as a barrier that restricts the dispersal of pelagic larvae (gene flow) of *L. brevicula* in winter. The source of the newly colonized populations of *L. brevicula* on the artificial structures of the YRD was highly consistent with the recent discoveries about the coastal currents in the studied area (Wu et al., 2018). For decades, a southward coastal current, the Yellow Sea Coastal Current, was considered to exist in the inner southwestern Yellow Sea during the wintertime Eastern Asian Monsoon (Guan, 1984; Yuan & Hsueh, 2010), which were also widely accepted by marine ecologists and also molecular ecologists (Guo et al., 2020; Ni et al., 2017; Wang et al., 2020; Zhou, 2010). Considering the wintertime spawning season of *L. brevicula* and the 33.5°N as a boundary that restricts the gene flow from north to south, the long‐term hypothesis of the southward Yellow Sea Coastal Current was clearly not supported by our genetic evidence. However, recent in situ observational evidence on two nearby stations demonstrated that the subtidal transport in the coastal water of inner southwestern Yellow Sea is also northward in wintertime. Further numerical experiments illustrated that this northward coastal current (the Subei tide‐induced coastal current (STCC)) exists from the YRE all the way along the Subei Coast until reaching ∼33.5°N; thereafter, it converges with southward coastal current from Haizhou Bay and turns offshore gradually in the vicinity of the Old Yellow River Delta (Wu et al., 2018). As a winter‐spawning species, the southern origin of the newly colonized populations on the Subei shoal strongly supported the northward wintertime coastal current in the inner southwestern Yellow Sea as suggested based on in situ observational evidence in Wu et al. (2018). Furthermore, the 33.5°N as a boundary that restricts the gene flow in *L. brevicula* was also perfectly consistent with the boundary of the STCC based on numerical simulation in Wu et al. (2018). For the first time, our genetic evidence of a wintertime spawning rocky‐shore marine invertebrate clearly demonstrated a northward wintertime coastal current in the inner southwestern Yellow Sea, and confirmed the boundary of the coastal current that was proposed by numerical simulation.

A variety of ocean current patterns may prevent dispersal across an “invisible barrier” (Palumbi, 1994). For the *Littorina* species around Point Conception in California, model simulation results confirm that a consistent, convergent ocean current pattern can produce an effective barrier to gene flow (Hohenlohe, 2004). In the present study, the convergent ocean current pattern between STCC and the southward coastal current from Haizhou Bay around 33.5°N could also be an effective barrier to gene flow for *L. brevicula*. Such knowledge can ultimately be extended to other commercially and ecologically important marine species with similar distributions, spawning season, and larval life span in this region, which would have important implications for the management and conservation of the Yellow Sea ecosystem. Therefore, our genetic evidence clearly demonstrated that the YRE could also be the source area of materials and energy for the inner southwestern Yellow Sea in wintertime, rather than the opposite as previously thought.

Habitat availability, larval transport, and temperature are important factors controlling the range limits of coastal marine species (Gaylord & Gaines, 2000; Lonhart et al., 2019; Wang et al., 2020). Along the coast of southwestern Yellow Sea, Wang et al. (2020) proposed that minimum temperature in winter around 33°N is an essential limiting factor setting the new northern range limit of the southern hard‐shore species, which may continue to shift to higher latitudes with ongoing global change. In addition to temperature, ocean currents also play an important role in shaping range boundaries of species because the dispersal of most marine species occurs during a pelagic larval phase (Cowen & Sponaugle, 2009; Gaylord & Gaines, 2000). The “red line” for the northern range limit of southern hard‐shore species at 33° N is mostly consistent with the barrier at 33.5°N in our study. Two of the species in Wang et al. (2020) only occur to the south of the YRE and spawn in summer season. In summer season, the STCC should still move northward and could be even stronger, because the southerly wind drives the current in the same direction (Wu et al., 2018). Although the summer range of STCC is still not clear so far, if the STCC could traverse 33.5 °N in summer and transport the larvae further north, the minimum temperature in winter around 33°N could be a limiting factor for the hard‐shore species with natural distribution confined to the south of the YRE. However, if the summer northern boundary of STCC is about the same as that in winter and larvae could not be transported further north by STCC, the range of southern hard‐shore species could not shift to higher latitudes even if temperature rises. Here, we proposed that the coastal currents in the southwestern Yellow Sea should be an important limiting factor setting the northern range boundary of the southern hard‐shore species, especially for those spawning in winter season.

Understanding connectivity over different spatial and temporal scales is fundamental for managing of ecological systems (Jolly et al., 2009; Pineda et al., 2018; Virtanen et al., 2020). In the present study, by precisely pinpointing the source of the newly colonized populations of *L. brevicula* on the artificial structures along coastal areas of the YRD, we clearly demonstrate strong ecological connectivity between the inner southwestern Yellow Sea and the YRE in winter time. For the first time, we presented genetic evidence that support a northward wintertime coastal current in the inner southwestern Yellow Sea, and precisely confirmed the boundary of the coastal current recently proposed based on numerical simulation. The strong wintertime ecological connectivity between the inner southwestern Yellow Sea and the YRE indicates that the YRE serves as an important source of materials and energy for the inner southwestern Yellow Sea in winter season, which could be crucial for the function of the Yellow Sea ecosystem.

## CONFLICT OF INTEREST

The authors have no competing interests.

## AUTHOR CONTRIBUTION

**Yu‐Qiang Li:** Data curation (lead); Formal analysis (lead); Writing‐original draft (equal). **Meng‐Yu Li:** Data curation (supporting); Formal analysis (supporting); Writing‐original draft (supporting). **Teng‐Fei Xing:** Investigation (equal); Resources (equal). **Jin‐Xian Liu:** Conceptualization (lead); Funding acquisition (lead); Investigation (equal); Project administration (lead); Resources (lead); Supervision (lead); Writing‐review & editing (lead).

## Supporting information

Supplementary Material

## Data Availability

All the mitochondrial ND6 sequences have been submitted to the GenBank databases with accession IDs MZ062607–MZ062896. The microsatellite data that support the findings of this study are openly available in Figshare with https://doi.org/10.6084/m9.figshare.13726516
