# Supplementary material for: Resolving the origins of invertebrate colonists in the Yangtze River Estuary with molecular markers: Implications for ecological connectivity
Source: Ecol Evol. 2021 Sep 16;11(20):13898–911. doi: 10.1002/ece3.8095 (PMC8525129; doi:10.1002/ece3.8095)
Supplement: Supplementary file 1 — Supplementary Material [file ECE3-11-13898-s001.pdf]

Resolving the origins of invertebrate colonists in the Yangtze River Estuary with molecular markers: implications for ecological connectivity

Supporting Document

Yu-Qiang Li[1,4], Meng-Yu Li[1,4], Teng-Fei Xing[1,4], Jin-Xian Liu[1,2,3,*]

[1] CAS Key Laboratory of Marine Ecology and Environmental Sciences, Institute of Oceanology, Chinese Academy of Sciences, Qingdao 266071, China.

[2] Laboratory for Marine Ecology and Environmental Science, Qingdao National Laboratory for Marine Science and Technology, Qingdao 266237, China.

[3] Center for Ocean Mega-Science, Chinese Academy of Sciences, Qingdao 266071, China.

[4] University of Chinese Academy of Sciences, Beijing 100049, China.

[*] **Corresponding author**: Jin-Xian Liu, **Email**: jinxianliu@gmail.com

Supplementary Tables and Figures

■ Tables

Table S1: Detail of 12 polymorphic microsatellite loci.

Locus	Primer sequences 5'-3'	Repeat motif	Ta (℃)	Size
Lbr10	F:GCGATAGGTCCCATTGCCAA	(AAC)21	53	287
	R:ACTCTCATGGATGTGAGCCC			
Lbr15	F:ACGTCTTTATCATGCGGCCT	(AAC)14	53	278
	R:AGCTATCGAAACTTTGGGTATGC			
Lbr21	F:CTAGAGAAGTCCCGTGCAGC	(AAAG)11	53	147
	R:GGACTGACCGACATGTAACCA			
Lbr25	F:TGAGTCCAGTGCCATTTCACA	(AAC)14	53	136
	R:TGCGGACAGGGATGTTACAAA			
Lbr31	F:ACGGACGTACTTGTCTGAATGT	(AC)10	53	197
	R:TTGCGTTTGTGAACTGACCG			
Lbr32	F:TGTATGCGCATGTGACTCGT	(AC)10	58	215
	R:ATCACTTGCACATTTCTACATAACA			
Lbr38	F:TGCTAAGTCAAAGTAATCAAGAATCCA	(AG)10	58	308
	R:TGGATAGTAGCAGTTGAGATGTTCT			
Lbr40	F:ACCGTTCTCTGACAAGTACACA	(AG)10	58	315
	R:GGTGCATCAATGAAACCAATGT			
Lbr41	F:TGTGACTCATGCAGTTCCCG	(AC)12	56	222
	R:CAAGACCGGTTTCGACGACT			
Lbr43	F:CCTGTAGTGTGGCAAAGGGA	(AC)10	56	214
	R:TACGCCTAGTCCCGAAATGC			
Lbr44	F:ATCACAACTGTGTACGGGTGA	(AC)10	56	290
	R:ACTGCGTCTGCCAATGAAAC			
Lbr46	F:GACCATGTTGGCTTGGCAAG	(AG)14	56	231
	R:CCTGTCTTGCTAGGTGAGAGC			

Table S2: Distribution of mitochondrial ND6 haplotypes among 12 populations for *Littorina brevicula* on the coast of Yangtze River Delta. Haplotype name is abbreviated as H1 to H54.

H_ID	QD	RZ	LYG	ZZ	SYG	DFG	YGD	LS	JQD	YDG	ZS	LH	Total
H1	21	22	20	18	18	1	1	0	0	4	0	2	107
H2	1	0	0	0	0	0	0	0	0	0	0	0	1
H3	1	0	0	0	0	0	0	0	0	0	0	0	1
H4	1	0	0	1	0	0	0	0	0	0	0	0	2
H5	0	1	0	0	0	0	0	0	0	0	0	0	1
H6	0	1	0	0	0	0	0	0	0	0	0	0	1
H7	0	0	1	0	0	0	0	0	0	0	0	0	1
H8	0	0	1	0	0	0	0	0	0	0	0	0	1
H9	0	0	1	0	0	0	0	0	0	0	0	0	1
H10	0	0	1	0	0	0	0	0	0	0	0	0	1
H11	0	0	0	1	0	0	0	0	0	0	0	0	1
H12	0	0	0	1	0	0	0	0	0	0	0	0	1
H13	0	0	0	1	0	0	0	0	0	0	0	0	1
H14	0	0	0	1	0	0	0	0	0	0	0	0	1
H15	0	0	0	1	0	0	0	0	0	0	0	0	1
H16	0	0	0	0	1	0	0	0	0	0	0	0	1
H17	0	0	0	0	1	0	0	0	0	0	0	0	1
H18	0	0	0	0	1	0	0	0	0	0	0	0	1
H19	0	0	0	0	1	0	0	0	0	0	0	0	1
H20	0	0	0	0	1	0	0	0	0	0	0	0	1
H21	0	0	0	0	1	2	5	2	2	5	3	2	22
H22	0	0	0	0	0	14	7	8	10	10	13	10	72
H23	0	0	0	0	0	2	4	5	1	3	1	2	18
H24	0	0	0	0	0	1	0	0	0	0	0	0	1
H25	0	0	0	0	0	2	1	3	2	0	2	1	11
H26	0	0	0	0	0	1	0	0	0	0	0	0	1
H27	0	0	0	0	0	1	0	0	0	0	0	0	1
H28	0	0	0	0	0	0	1	0	0	0	0	0	1
H29	0	0	0	0	0	0	2	0	0	0	0	0	2
H30	0	0	0	0	0	0	1	0	0	0	0	0	1
H31	0	0	0	0	0	0	1	0	0	0	0	0	1
H32	0	0	0	0	0	0	1	0	0	0	0	0	1
H33	0	0	0	0	0	0	0	6	1	0	0	1	8
H34	0	0	0	0	0	0	0	1	0	0	0	0	1
H35	0	0	0	0	0	0	0	0	0	1	0	0	1
H36	0	0	0	0	0	0	0	0	0	1	0	0	1
H37	0	0	0	0	0	0	0	0	1	0	0	0	1
H38	0	0	0	0	0	0	0	0	1	0	0	0	1
H39	0	0	0	0	0	0	0	0	1	0	0	0	1
H40	0	0	0	0	0	0	0	0	1	0	0	0	1

H41	0	0	0	0	0	0	0	0	1	0	0	0	1
H42	0	0	0	0	0	0	0	0	1	0	0	0	1
H43	0	0	0	0	0	0	0	0	2	0	0	1	3
H44	0	0	0	0	0	0	0	0	1	0	0	0	1
H45	0	0	0	0	0	0	0	0	0	0	1	0	1
H46	0	0	0	0	0	0	0	0	0	0	1	0	1
H47	0	0	0	0	0	0	0	0	0	0	1	0	1
H48	0	0	0	0	0	0	0	0	0	0	1	0	1
H49	0	0	0	0	0	0	0	0	0	0	1	0	1
H50	0	0	0	0	0	0	0	0	0	0	0	1	1
H51	0	0	0	0	0	0	0	0	0	0	0	1	1
H52	0	0	0	0	0	0	0	0	0	0	0	1	1
H53	0	0	0	0	0	0	0	0	0	0	0	1	1
H54	0	0	0	0	0	0	0	0	0	0	0	1	1

Table S3: The *p*-values of Hardy-Weinberg equilibrium test for 12 microsatellite loci within 12 populations for *Littorina brevicula* on the coast of Yangtze River Delta.

	Lbr10	Lbr15	Lbr21	Lbr25	Lbr31	Lbr32	Lbr38	Lbr40	Lbr41	Lbr43	Lbr44	Lbr46
QD	**0.000**	**0.000**	0.033	0.795	0.206	0.388	0.053	0.283	0.007	0.140	0.672	0.286
RZ	**0.000**	0.021	0.009	0.181	0.636	0.178	0.986	0.949	0.098	0.000	0.130	0.177
LYG	**0.000**	**0.000**	0.241	0.157	0.438	0.024	0.499	0.865	0.005	0.000	0.329	0.166
ZDZ	**0.000**	**0.000**	0.005	0.258	0.729	0.469	0.682	0.849	**0.000**	0.012	0.079	1.000
SYG	**0.000**	**0.000**	0.063	0.990	0.076	0.171	0.465	0.950	**0.000**	0.003	0.030	0.423
DFG	**0.000**	**0.000**	0.034	**0.000**	0.109	**0.000**	0.500	0.045	**0.000**	0.022	0.405	0.105
YGD	**0.000**	**0.000**	0.090	0.019	0.176	**0.000**	1.000	0.036	**0.000**	0.578	0.222	0.326
LS	**0.002**	**0.000**	0.619	**0.000**	0.517	**0.001**	1.000	0.155	**0.000**	**0.000**	0.703	0.307
JQD	**0.000**	**0.000**	**0.001**	**0.000**	0.071	0.007	1.000	0.004	**0.000**	0.021	0.965	0.066
YSG	0.004	**0.000**	0.300	**0.000**	0.428	0.191	1.000	0.816	**0.000**	0.118	0.244	0.821
ZS	**0.000**	**0.000**	0.443	**0.001**	0.434	0.031	1.000	0.079	**0.000**	0.076	0.004	0.545
LH	**0.000**	**0.000**	0.479	**0.000**	0.165	0.053	0.244	0.849	**0.000**	**0.000**	0.371	0.441

† Significant deviation from Hardy-Weinberg equilibrium (*p*-value <0.004, after Bonferroni corrected correction) are indicated in bold.

Table S4：The *p*-values of linkage-disequilibrium test for each locus pair across 12 populations for *Littorina brevicula* on the coast of Yangtze River Delta.

Locus pair	QD	RZ	LYG	ZDZ	SYG	DFG	YGD	LS	JQD	YSG	ZS	LH
Lbr10&Lbr15	0.393	1.000	1.000	1.000	1.000	1.000	0.565	1.000	0.283	1.000	1.000	1.000
Lbr10&Lbr21	1.000	1.000	1.000	0.394	1.000	1.000	0.255	1.000	0.165	1.000	1.000	1.000
Lbr15&Lbr21	0.658	1.000	0.496	0.024	1.000	1.000	0.322	1.000	1.000	1.000	1.000	1.000
Lbr10&Lbr25	1.000	1.000	1.000	1.000	0.117	1.000	0.177	1.000	1.000	1.000	1.000	1.000
Lbr15&Lbr25	1.000	0.354	0.265	1.000	0.135	1.000	0.208	1.000	1.000	1.000	1.000	1.000
Lbr21&Lbr25	1.000	1.000	0.358	1.000	1.000	0.130	0.156	1.000	1.000	1.000	1.000	0.081
Lbr10&Lbr31	0.593	1.000	0.425	1.000	1.000	0.782	0.456	0.024	0.268	1.000	0.053	1.000
Lbr15&Lbr31	0.156	0.370	0.592	0.198	0.482	0.922	0.680	0.463	0.924	0.623	0.842	0.262
Lbr21&Lbr31	0.322	0.105	0.119	0.872	0.530	0.484	0.027	1.000	0.707	1.000	0.227	1.000
Lbr25&Lbr31	1.000	1.000	0.346	1.000	0.755	1.000	0.199	0.693	0.160	0.605	0.645	0.633
Lbr10&Lbr32	0.383	1.000	0.289	1.000	1.000	0.432	0.644	0.386	0.019	0.793	0.578	1.000
Lbr15&Lbr32	0.628	0.767	1.000	1.000	**0.000**	0.147	0.015	1.000	0.701	1.000	0.906	0.885
Lbr21&Lbr32	0.203	0.827	0.495	1.000	1.000	0.592	0.351	1.000	0.424	0.256	1.000	1.000
Lbr25&Lbr32	1.000	0.688	1.000	1.000	0.350	0.530	0.637	0.501	1.000	0.547	0.823	0.425
Lbr31&Lbr32	0.913	0.931	0.394	0.892	0.304	0.427	0.726	0.969	0.530	1.000	0.017	0.877
Lbr10&Lbr38	0.491	1.000	1.000	0.049	1.000	0.271	0.516	1.000	0.964	0.796	0.608	1.000
Lbr15&Lbr38	1.000	1.000	1.000	0.215	0.618	0.216	0.280	0.471	0.325	0.464	0.557	0.281
Lbr21&Lbr38	0.074	1.000	0.527	0.204	1.000	0.470	0.217	1.000	0.501	0.328	0.691	0.602

Lbr25&Lbr38	0.636	1.000	1.000	1.000	0.285	0.070	0.415	0.928	0.704	0.875	0.664	0.359
Lbr31&Lbr38	0.532	0.700	0.633	1.000	0.996	0.912	0.349	0.042	0.971	0.483	0.003	0.037
Lbr32&Lbr38	0.916	1.000	0.786	0.596	0.277	0.663	0.432	0.916	0.438	1.000	0.191	0.886
Lbr10&Lbr40	1.000	1.000	1.000	1.000	1.000	1.000	0.111	1.000	0.379	1.000	1.000	1.000
Lbr15&Lbr40	1.000	1.000	0.510	1.000	1.000	0.052	0.701	1.000	1.000	0.276	0.690	0.391
Lbr21&Lbr40	1.000	1.000	0.188	0.276	1.000	0.691	0.255	1.000	0.383	0.389	0.168	1.000
Lbr25&Lbr40	0.357	1.000	0.253	0.141	1.000	1.000	0.451	1.000	0.625	0.272	1.000	1.000
Lbr31&Lbr40	1.000	0.259	0.227	0.970	0.334	0.536	0.370	0.417	0.137	0.992	0.654	0.618
Lbr32&Lbr40	0.736	0.977	0.726	0.272	1.000	0.512	0.959	0.448	0.867	0.998	0.997	0.425
Lbr38&Lbr40	0.179	0.559	0.878	0.680	0.083	0.264	0.244	0.639	0.618	0.214	0.678	0.481
Lbr10&Lbr41	1.000	1.000	1.000	1.000	1.000	1.000	0.278	1.000	1.000	1.000	1.000	1.000
Lbr15&Lbr41	0.788	1.000	1.000	1.000	0.509	0.232	0.052	1.000	0.442	1.000	1.000	1.000
Lbr21&Lbr41	1.000	1.000	1.000	1.000	0.282	1.000	0.129	1.000	1.000	1.000	1.000	0.178
Lbr25&Lbr41	1.000	1.000	1.000	1.000	1.000	1.000	0.116	1.000	1.000	1.000	0.583	1.000
Lbr31&Lbr41	0.815	1.000	0.796	0.897	0.089	0.040	0.498	1.000	0.508	1.000	0.402	0.925
Lbr32&Lbr41	0.209	0.612	0.063	0.636	0.789	0.671	0.382	1.000	1.000	0.749	0.864	0.704
Lbr38&Lbr41	1.000	0.143	0.329	1.000	1.000	0.408	0.715	0.873	0.919	1.000	0.925	0.682
Lbr40&Lbr41	0.555	1.000	1.000	0.799	1.000	0.466	0.675	1.000	1.000	0.372	0.293	0.573
Lbr10&Lbr43	0.546	0.301	0.582	0.140	0.393	0.831	0.350	1.000	0.526	0.623	1.000	0.574
Lbr15&Lbr43	0.961	0.862	0.522	0.853	0.693	0.971	0.656	0.513	0.651	0.877	0.698	0.916

Lbr21&Lbr43	0.408	0.646	0.334	0.598	1.000	0.819	0.039	1.000	0.907	0.934	1.000	1.000
Lbr25&Lbr43	0.244	0.970	0.601	0.612	1.000	0.800	0.248	0.419	0.898	0.890	0.272	0.922
Lbr31&Lbr43	0.292	0.860	0.303	0.818	0.909	0.249	0.727	0.435	0.447	0.663	0.632	0.408
Lbr32&Lbr43	0.509	0.503	0.707	0.596	0.875	0.716	0.285	0.118	0.057	0.797	0.623	0.163
Lbr38&Lbr43	0.163	0.970	0.517	0.279	0.645	0.923	0.803	0.329	0.325	0.268	1.000	0.819
Lbr40&Lbr43	0.705	0.995	0.108	0.059	0.984	0.961	0.669	0.862	0.978	0.165	0.899	0.597
Lbr41&Lbr43	0.394	0.901	0.634	0.907	0.924	0.954	0.953	0.378	0.972	0.554	0.655	0.411
Lbr10&Lbr44	0.568	0.135	0.168	0.268	0.085	0.165	0.063	1.000	1.000	1.000	1.000	1.000
Lbr15&Lbr44	0.033	0.718	0.510	0.380	0.868	1.000	0.085	1.000	0.335	0.133	1.000	1.000
Lbr21&Lbr44	0.727	0.280	0.513	0.879	0.558	1.000	0.253	1.000	1.000	1.000	0.157	1.000
Lbr25&Lbr44	0.738	0.172	1.000	1.000	0.083	1.000	0.119	0.078	0.302	1.000	0.301	1.000
Lbr31&Lbr44	0.774	0.574	0.092	0.928	0.573	0.439	0.945	0.603	0.693	0.702	0.089	0.508
Lbr32&Lbr44	0.177	0.004	1.000	0.960	0.909	0.177	0.028	1.000	1.000	0.759	0.801	0.597
Lbr38&Lbr44	0.502	1.000	0.084	0.234	0.124	0.672	0.443	0.907	0.122	0.699	0.805	0.980
Lbr40&Lbr44	0.309	0.688	0.573	0.314	0.285	0.626	0.130	1.000	0.394	0.016	0.333	1.000
Lbr41&Lbr44	0.874	0.029	1.000	0.558	0.161	0.225	0.020	1.000	1.000	0.150	0.143	1.000
Lbr43&Lbr44	0.941	0.923	0.804	0.106	0.298	0.553	0.854	0.865	0.788	0.909	0.763	0.651
Lbr10&Lbr46	0.795	0.468	1.000	0.868	0.529	0.698	0.266	1.000	0.708	0.232	1.000	1.000
Lbr15&Lbr46	0.696	0.936	0.404	0.151	1.000	0.672	0.286	0.903	0.330	1.000	0.917	0.281
Lbr21&Lbr46	0.631	0.743	0.251	0.763	1.000	0.099	0.058	1.000	0.718	0.868	1.000	0.109

Lbr25&Lbr46	0.254	0.567	0.612	1.000	0.377	0.153	0.613	0.706	0.939	0.794	0.666	0.515
Lbr31&Lbr46	0.355	0.192	0.520	0.495	0.070	0.876	0.087	0.756	0.505	0.009	0.116	0.594
Lbr32&Lbr46	0.618	0.342	0.753	0.503	0.362	0.637	0.689	0.498	0.627	0.690	0.426	0.521
Lbr38&Lbr46	0.967	0.549	0.444	0.543	0.069	0.749	0.049	0.505	0.123	0.760	0.487	0.118
Lbr40&Lbr46	0.152	0.919	0.716	0.317	0.096	0.754	0.573	0.986	0.580	0.752	0.611	0.699
Lbr41&Lbr46	0.970	0.801	1.000	0.036	0.544	0.929	0.453	1.000	0.525	0.851	0.335	0.827
Lbr43&Lbr46	0.718	0.232	0.611	0.907	0.673	0.721	0.804	0.564	0.142	0.485	0.344	0.808
Lbr44&Lbr46	0.986	0.386	0.504	0.281	0.031	0.968	0.930	1.000	0.072	0.659	0.212	0.355

† Significant deviation from linkage disequilibrium (*p*-value <0.0008, after Bonferroni corrected correction) are indicated in bold.

Table S5: Results of the null alleles test for 12 microsatellite loci within 12 populations for *Littorina brevicula* on the coast of Yangtze River Delta.

Loci	QD	RZ	LYG	ZDZ	SYG	DFG	YGD	LS	JQD	YSG	ZS	LH
Lbr10	yes	yes	yes	yes	yes	yes	yes	yes	yes	yes	yes	yes
Lbr15	yes	yes	yes	yes	yes	yes	yes	yes	yes	yes	yes	yes
Lbr21	yes	yes	no	no	yes	yes	no	no	yes	no	no	no
Lbr25	no	no	no	no	no	yes	yes	yes	yes	yes	yes	yes
Lbr31	no	no	no	no	no	no	no	no	no	no	no	no
Lbr32	no	no	no	no	no	yes	yes	yes	yes	no	yes	no
Lbr38	no	no	no	no	no	no	no	no	no	no	no	no
Lbr40	no	no	no	no	no	yes	yes	no	yes	no	no	no
Lbr41	yes	no	yes	yes	yes	yes	yes	yes	yes	yes	yes	yes
Lbr43	no	yes	yes	yes	yes	yes	no	yes	yes	no	yes	yes
Lbr44	no	no	no	no	no	no	no	no	no	no	yes	no
Lbr46	no	no	no	no	no	no	no	no	no	no	no	no

Table S6: Summary of the statistics for 12 microsatellite loci within 12 populations for *Littorina brevicula* on the coast of Yangtze River Delta.

Locality		Lbr10	Lbr15	Lbr21	Lbr25	Lbr31	Lbr32	Lbr38	Lbr40	Lbr41	Lbr43	Lbr44	Lbr46
QD	H_O	0.52	0.33	0.61	0.88	0.61	0.67	0.63	0.83	0.50	0.29	0.63	0.71
	H_E	0.89	0.83	0.84	0.83	0.72	0.77	0.75	0.82	0.82	0.42	0.66	0.61
	PIC	0.86	0.79	0.80	0.80	0.65	0.71	0.70	0.78	0.76	0.39	0.61	0.56
	AR	14.78	10.73	6.96	11.73	4.96	5.92	7.83	8.83	9.87	4.83	5.99	5.98
	*N*a	15.00	11.00	7.00	12.00	5.00	6.00	8.00	9.00	10.00	5.00	6.00	6.00
	F_{IS}	0.42	0.60	0.28	-0.05	0.16	0.13	0.17	-0.02	0.36	0.32	0.05	-0.16
RZ	H_O	0.46	0.58	0.58	0.71	0.58	0.63	0.79	0.75	0.88	0.21	0.67	0.50
	H_E	0.91	0.84	0.85	0.85	0.70	0.72	0.81	0.72	0.90	0.57	0.72	0.59
	PIC	0.88	0.80	0.82	0.81	0.62	0.66	0.77	0.67	0.85	0.50	0.69	0.52
	AR	13.73	8.83	8.91	10.66	4.83	5.83	9.73	6.90	11.95	3.92	10.81	5.83
	*N*a	14.00	9.00	9.00	11.00	5.00	6.00	10.00	7.00	12.00	4.00	11.00	6.00
	F_{IS}	0.50	0.31	0.32	0.17	0.17	0.13	0.03	-0.04	0.07	0.64	0.07	0.16
LYG	H_O	0.63	0.33	0.63	0.83	0.71	0.71	0.88	0.79	0.65	0.13	0.79	0.54
	H_E	0.93	0.90	0.79	0.88	0.69	0.77	0.84	0.70	0.88	0.36	0.82	0.69
	PIC	0.90	0.87	0.75	0.85	0.62	0.71	0.80	0.63	0.85	0.33	0.78	0.63
	AR	12.83	14.57	8.74	13.49	4.00	5.00	8.99	5.75	12.82	3.92	11.57	5.91
	*N*a	13.00	15.00	9.00	14.00	4.00	5.00	9.00	6.00	13.00	4.00	12.00	6.00
	F_{IS}	0.33	0.64	0.22	0.06	-0.02	0.08	-0.05	-0.13	0.26	0.66	0.03	0.22

ZDZ	H_O	0.35	0.33	0.70	0.75	0.79	0.59	0.83	0.83	0.48	0.26	0.50	0.58
	H_E	0.92	0.91	0.84	0.89	0.72	0.77	0.82	0.78	0.81	0.44	0.63	0.51
	PIC	0.89	0.88	0.80	0.86	0.67	0.71	0.77	0.73	0.76	0.40	0.59	0.47
	AR	12.91	14.65	7.96	12.66	5.83	6.00	7.96	8.74	6.96	4.00	6.91	4.99
	*N*a	13.00	15.00	8.00	13.00	6.00	6.00	8.00	9.00	7.00	4.00	7.00	5.00
	F_{IS}	0.63	0.64	0.17	0.16	-0.10	0.24	-0.01	-0.07	0.47	0.41	0.22	-0.15
SYG	H_O	0.54	0.42	0.67	0.96	0.67	0.65	0.77	0.88	0.54	0.33	0.57	0.63
	H_E	0.92	0.88	0.87	0.88	0.59	0.75	0.78	0.78	0.86	0.58	0.71	0.69
	PIC	0.89	0.84	0.83	0.85	0.51	0.70	0.72	0.74	0.82	0.51	0.67	0.62
	AR	13.73	16.22	9.91	14.48	3.00	5.00	7.00	8.75	9.74	3.92	8.91	5.83
	*N*a	14.00	17.00	10.00	15.00	3.00	5.00	7.00	9.00	10.00	4.00	9.00	6.00
	F_{IS}	0.42	0.53	0.24	-0.09	-0.14	0.14	0.01	-0.12	0.42	0.43	0.20	0.09
DFG	H_O	0.50	0.42	0.67	0.75	0.63	0.30	0.33	0.63	0.58	0.25	0.79	0.21
	H_E	0.91	0.91	0.85	0.94	0.69	0.65	0.34	0.77	0.90	0.43	0.85	0.30
	PIC	0.88	0.88	0.81	0.91	0.62	0.58	0.32	0.74	0.85	0.39	0.81	0.27
	AR	12.74	15.63	9.82	16.57	4.00	5.00	5.75	9.74	9.91	4.91	10.73	3.91
	*N*a	13.00	16.00	10.00	17.00	4.00	5.00	6.00	10.00	10.00	5.00	11.00	4.00
	F_{IS}	0.46	0.55	0.22	0.20	0.10	0.54	0.01	0.20	0.42	0.42	0.07	0.31
YGD	H_O	0.54	0.38	0.75	0.74	0.67	0.42	0.25	0.46	0.63	0.29	0.96	0.54
	H_E	0.88	0.87	0.88	0.91	0.62	0.67	0.23	0.66	0.91	0.33	0.89	0.59

	PIC	0.85	0.84	0.85	0.88	0.54	0.63	0.21	0.60	0.87	0.31	0.86	0.52
	AR	12.65	10.82	9.75	14.74	5.75	6.83	2.92	6.75	9.91	4.83	15.48	5.83
	*N*a	13.00	11.00	10.00	15.00	6.00	7.00	3.00	7.00	10.00	5.00	16.00	6.00
	F_{IS}	0.39	0.57	0.15	0.19	-0.07	0.39	-0.10	0.32	0.45	0.13	-0.08	0.09
LS	H_O	0.63	0.21	0.88	0.57	0.58	0.52	0.42	0.71	0.58	0.25	0.75	0.46
	H_E	0.91	0.92	0.92	0.91	0.64	0.77	0.37	0.80	0.93	0.58	0.85	0.57
	PIC	0.88	0.90	0.89	0.88	0.58	0.71	0.35	0.76	0.90	0.53	0.82	0.52
	AR	15.32	17.62	11.91	13.91	4.91	6.95	6.67	11.42	12.91	5.83	11.57	6.82
	*N*a	16.00	18.00	12.00	14.00	5.00	7.00	7.00	12.00	13.00	6.00	12.00	7.00
	F_{IS}	0.32	0.78	0.05	0.38	0.09	0.33	-0.13	0.12	0.35	0.57	0.12	0.20
JQD	H_O	0.50	0.29	0.58	0.38	0.54	0.48	0.25	0.58	0.33	0.30	0.83	0.46
	H_E	0.92	0.92	0.89	0.88	0.66	0.75	0.23	0.80	0.89	0.55	0.84	0.65
	PIC	0.90	0.90	0.86	0.85	0.60	0.71	0.22	0.75	0.86	0.48	0.80	0.58
	AR	15.48	16.55	10.74	12.58	4.92	8.91	4.82	9.58	10.82	4.91	10.66	6.74
	*N*a	16.00	17.00	11.00	13.00	5.00	9.00	5.00	10.00	11.00	5.00	11.00	7.00
	F_{IS}	0.47	0.69	0.35	0.58	0.18	0.37	-0.07	0.27	0.63	0.45	0.01	0.30
YSG	H_O	0.71	0.42	0.79	0.54	0.79	0.41	0.13	0.71	0.54	0.29	0.79	0.54
	H_E	0.89	0.93	0.89	0.91	0.64	0.56	0.13	0.74	0.94	0.42	0.84	0.52
	PIC	0.86	0.90	0.86	0.89	0.58	0.51	0.12	0.68	0.89	0.37	0.80	0.43
	AR	14.49	16.48	10.83	13.81	4.92	5.00	2.96	5.92	13.00	4.83	9.74	4.75

	*N*a	15.00	17.00	11.00	14.00	5.00	5.00	3.00	6.00	13.00	5.00	10.00	5.00
	F_{IS}	0.21	0.56	0.11	0.41	-0.24	0.28	-0.03	0.05	0.56	0.30	0.06	-0.04
ZS	H_O	0.42	0.50	0.83	0.67	0.58	0.39	0.08	0.71	0.42	0.33	0.67	0.46
	H_E	0.94	0.90	0.91	0.89	0.69	0.62	0.08	0.77	0.88	0.51	0.85	0.46
	PIC	0.92	0.87	0.89	0.86	0.62	0.55	0.08	0.72	0.81	0.47	0.81	0.44
	AR	16.72	11.82	12.82	12.73	4.83	4.00	2.83	9.66	9.91	5.83	9.74	7.58
	*N*a	17.00	12.00	13.00	13.00	5.00	4.00	3.00	10.00	10.00	6.00	10.00	8.00
	F_{IS}	0.56	0.45	0.09	0.26	0.16	0.38	-0.01	0.08	0.71	0.35	0.22	0.01
LH	H_O	0.48	0.13	0.79	0.54	0.63	0.57	0.29	0.75	0.50	0.22	0.79	0.67
	H_E	0.90	0.88	0.88	0.92	0.65	0.74	0.37	0.75	0.89	0.55	0.85	0.68
	PIC	0.87	0.85	0.85	0.89	0.58	0.68	0.35	0.71	0.85	0.50	0.81	0.63
	AR	12.87	10.97	10.90	14.65	4.83	5.96	5.82	10.49	11.58	5.91	11.57	9.57
	*N*a	13.00	11.00	11.00	15.00	5.00	6.00	6.00	11.00	12.00	6.00	12.00	10.00
	F_{IS}	0.48	0.86	0.11	0.42	0.05	0.24	0.22	0.00	0.49	0.61	0.07	0.02

† Measures of genetic variations including: average observed heterozygosity (H_O), average expected heterozygosity (H_E), polymorphic information content (*PIC*), mean number of alleles per locus (*N*a), allelic richness (*AR*) and inbreeding coefficient (F_{IS})

Table S7: Pairwise F_{ST} values and the corresponding *p*-values between populations based on eight microsatellite loci for *Littorina brevicula* on the coast of Yangtze River Delta.

	QD	RZ	LYG	ZDZ	SYG	DFG	YGD	LS	JQD	YSG	ZS	LH
QD		0.606	0.144	0.119	0.038	0.000	0.000	0.000	0.000	0.000	0.000	0.000
RZ	0.000		0.106	0.383	0.638	0.000	0.000	0.000	0.000	0.000	0.000	0.000
LYG	0.007	0.009		0.142	0.070	0.000	0.000	0.000	0.000	0.000	0.000	0.000
ZDZ	0.008	0.003	0.007		0.250	0.000	0.000	0.000	0.000	0.000	0.000	0.000
SYG	0.012	-0.001	0.010	0.004		0.000	0.000	0.000	0.000	0.000	0.000	0.000
DFG	**0.115**	**0.111**	**0.117**	**0.106**	**0.128**		0.007	0.064	0.000	0.078	0.695	0.106
YGD	**0.146**	**0.150**	**0.144**	**0.155**	**0.166**	0.022		0.033	0.090	0.238	0.074	0.312
LS	**0.105**	**0.099**	**0.098**	**0.104**	**0.109**	0.014	0.015		0.335	0.107	0.283	0.962
JQD	**0.123**	**0.120**	**0.114**	**0.125**	**0.133**	**0.037**	0.013	0.007		0.399	0.039	0.339
YSG	**0.138**	**0.136**	**0.135**	**0.138**	**0.152**	0.013	0.006	0.010	0.005		0.497	0.321
ZS	**0.128**	**0.127**	**0.129**	**0.125**	**0.145**	0.000	0.013	0.006	0.018	0.002		0.451
LH	**0.103**	**0.103**	**0.103**	**0.107**	**0.114**	0.011	0.005	-0.006	0.006	0.005	0.003	

† F_{ST} values are shown below the diagonal, upper diagonal represents the *p*-values. Pairwise F_{ST} with significant p-values (*p*-value <0.0008, after Bonferroni corrected correction) are indicated in bold.

Table S8: Results of assignment and exclusion tests of the samples from newly colonized populations to the northern natural populations (NNP) and southern natural populations (SNP) based on 12 microsatellite loci.

	Assignment of individuals Threhold:0.05		Exclusion of individuals Threhold: >0.01 can't be excluded as source population	
Sample	RANK1	SCORE [%]	NNP probability	SNP probability
ZDZ01	NNP	99.78	0.01	0.00
ZDZ02	NNP	100.00	0.48	0.00
ZDZ03	NNP	99.98	0.52	0.11
ZDZ04	NNP	60.37	0.02	0.06
ZDZ05	NNP	99.99	0.08	0.01
ZDZ06	SNP	99.12	0.01	0.07
ZDZ07	NNP	100.00	0.62	0.04
ZDZ08	NNP	100.00	0.03	0.00
ZDZ09	NNP	100.00	0.44	0.01
ZDZ10	NNP	99.73	0.05	0.02
ZDZ11	NNP	100.00	0.77	0.00
ZDZ12	NNP	100.00	0.63	0.10
ZDZ13	NNP	100.00	0.92	0.01
ZDZ14	NNP	100.00	0.53	0.00
ZDZ15	NNP	100.00	0.64	0.00
ZDZ16	NNP	100.00	0.26	0.00
ZDZ17	NNP	99.97	0.06	0.01
ZDZ18	NNP	100.00	0.64	0.03
ZDZ19	NNP	100.00	0.81	0.00
ZDZ20	NNP	100.00	0.08	0.00
ZDZ21	NNP	100.00	0.35	0.00
ZDZ22	NNP	100.00	0.02	0.00
ZDZ23	NNP	100.00	0.55	0.00
ZDZ24	NNP	100.00	0.65	0.04
SYG01	NNP	100.00	0.56	0.01
SYG02	NNP	99.86	0.15	0.04
SYG03	NNP	98.25	0.01	0.00

SYG04	NNP	100.00	0.77	0.02
SYG05	NNP	100.00	0.09	0.00
SYG06	NNP	100.00	0.62	0.00
SYG07	NNP	100.00	0.57	0.00
SYG08	NNP	100.00	0.02	0.00
SYG09	NNP	100.00	0.22	0.00
SYG10	NNP	100.00	0.89	0.01
SYG11	NNP	100.00	0.12	0.00
SYG12	NNP	100.00	0.54	0.00
SYG13	NNP	100.00	0.85	0.00
SYG14	NNP	100.00	0.09	0.00
SYG15	NNP	99.99	0.04	0.00
SYG16	NNP	77.33	0.13	0.25
SYG17	NNP	100.00	0.04	0.00
SYG18	NNP	100.00	0.15	0.00
SYG19	NNP	100.00	0.04	0.00
SYG20	NNP	100.00	0.41	0.01
SYG21	NNP	100.00	0.09	0.00
SYG22	NNP	100.00	0.44	0.04
SYG23	NNP	100.00	0.45	0.00
SYG24	NNP	100.00	0.20	0.00
DFG01	SNP	95.03	0.08	0.22
DFG02	SNP	100.00	0.00	0.21
DFG03	SNP	100.00	0.01	0.32
DFG04	SNP	100.00	0.00	0.68
DFG05	NNP	98.16	0.48	0.30
DFG06	SNP	100.00	0.00	0.12
DFG07	SNP	100.00	0.01	0.45
DFG08	SNP	100.00	0.00	0.16
DFG09	SNP	99.97	0.01	0.14
DFG10	SNP	100.00	0.00	0.40
DFG11	SNP	100.00	0.07	0.96
DFG12	SNP	100.00	0.00	0.17
DFG13	SNP	100.00	0.00	0.13
DFG14	SNP	100.00	0.00	0.27

DFG15	SNP	99.98	0.02	0.25
DFG16	SNP	100.00	0.00	0.73
DFG17	SNP	100.00	0.00	0.58
DFG18	SNP	100.00	0.00	0.52
DFG19	SNP	100.00	0.00	0.17
DFG20	SNP	100.00	0.00	0.25
DFG21	SNP	99.25	0.11	0.42
DFG22	SNP	100.00	0.00	0.03
DFG23	SNP	99.97	0.03	0.30
DFG24	SNP	100.00	0.00	0.09
YGD01	SNP	100.00	0.00	0.22
YGD02	SNP	100.00	0.00	0.26
YGD03	SNP	99.71	0.00	0.06
YGD04	SNP	100.00	0.00	0.41
YGD05	SNP	100.00	0.00	0.45
YGD06	SNP	100.00	0.00	0.22
YGD07	SNP	100.00	0.00	0.17
YGD08	SNP	100.00	0.01	0.42
YGD09	SNP	100.00	0.00	0.68
YGD10	SNP	100.00	0.01	0.97
YGD11	SNP	100.00	0.00	0.29
YGD12	SNP	100.00	0.01	0.58
YGD13	SNP	100.00	0.00	0.35
YGD14	SNP	100.00	0.00	0.06
YGD15	SNP	100.00	0.00	0.06
YGD16	SNP	99.95	0.01	0.14
YGD17	SNP	99.29	0.17	0.54
YGD18	SNP	100.00	0.00	0.77
YGD19	SNP	100.00	0.00	0.53
YGD20	SNP	100.00	0.00	0.67
YGD21	SNP	100.00	0.00	0.38
YGD22	SNP	100.00	0.00	0.00
YGD23	SNP	100.00	0.00	0.38
YGD24	SNP	100.00	0.01	0.48
LS01	SNP	100.00	0.00	0.33

LS02	SNP	100.00	0.00	0.17
LS03	SNP	100.00	0.00	0.05
LS04	SNP	100.00	0.00	0.08
LS05	SNP	100.00	0.00	0.07
LS06	SNP	100.00	0.00	0.04
LS07	SNP	69.24	0.01	0.02
LS08	SNP	99.97	0.05	0.45
LS09	SNP	100.00	0.00	0.17
LS10	SNP	99.99	0.00	0.01
LS11	SNP	100.00	0.01	0.72
LS12	SNP	100.00	0.00	0.02
LS13	SNP	100.00	0.02	0.60
LS14	SNP	100.00	0.00	0.02
LS15	SNP	100.00	0.00	0.10
LS16	SNP	100.00	0.03	0.75
LS17	SNP	100.00	0.00	0.23
LS18	SNP	100.00	0.14	0.85
LS19	SNP	100.00	0.00	0.00
LS20	SNP	99.96	0.00	0.00
LS21	SNP	100.00	0.00	0.42
LS22	SNP	99.94	0.01	0.10
LS23	SNP	99.91	0.00	0.00
LS24	SNP	100.00	0.02	0.57
JQD01	SNP	100.00	0.00	0.17
JQD02	SNP	100.00	0.00	0.85
JQD03	SNP	100.00	0.00	0.04
JQD04	SNP	100.00	0.00	0.31
JQD05	SNP	97.03	0.16	0.41
JQD06	SNP	100.00	0.00	0.08
JQD07	SNP	100.00	0.00	0.08
JQD08	SNP	99.98	0.04	0.43
JQD09	SNP	100.00	0.00	0.16
JQD10	SNP	100.00	0.01	0.32
JQD11	SNP	100.00	0.00	0.29
JQD12	SNP	100.00	0.00	0.00

JQD13	SNP	100.00	0.00	0.55
JQD14	SNP	100.00	0.00	0.11
JQD15	SNP	100.00	0.00	0.08
JQD16	SNP	99.99	0.00	0.08
JQD17	SNP	100.00	0.00	0.00
JQD18	SNP	100.00	0.00	0.06
JQD19	SNP	100.00	0.00	0.23
JQD20	SNP	100.00	0.03	0.71
JQD21	SNP	100.00	0.00	0.52
JQD22	SNP	100.00	0.00	0.49
JQD23	SNP	99.93	0.00	0.03
JQD23	SNP	100.00	0.01	0.34

Table S9: Results of assignment and exclusion tests of the samples from newly colonized populations to the northern natural populations (NNP) and southern natural populations (SNP) based on eight microsatellite loci.

	Assignment of individuals Threhold:0.05		Exclusion of individuals Threhold:>0.01 can't be excluded as source population	
Sample	RANK1	SCORE [%]	NNP probability	SNP probability
ZDZ01	NNP	100.00	0.11	0.00
ZDZ02	NNP	100.00	0.24	0.00
ZDZ03	NNP	100.00	0.60	0.03
ZDZ04	SNP	66.82	0.00	0.01
ZDZ05	NNP	100.00	0.11	0.00
ZDZ06	SNP	79.37	0.00	0.02
ZDZ07	NNP	100.00	0.54	0.02
ZDZ08	NNP	99.99	0.18	0.01
ZDZ09	NNP	100.00	0.70	0.00
ZDZ10	NNP	98.58	0.01	0.00
ZDZ11	NNP	100.00	0.53	0.00
ZDZ12	NNP	100.00	0.54	0.02
ZDZ13	NNP	100.00	0.97	0.02
ZDZ14	NNP	100.00	0.82	0.00
ZDZ15	NNP	100.00	0.51	0.00
ZDZ16	NNP	100.00	0.68	0.00
ZDZ17	NNP	100.00	0.50	0.01
ZDZ18	NNP	100.00	0.60	0.03
ZDZ19	NNP	100.00	0.75	0.00
ZDZ20	NNP	100.00	0.49	0.01
ZDZ21	NNP	100.00	0.66	0.00
ZDZ22	NNP	100.00	0.10	0.00
ZDZ23	NNP	100.00	0.47	0.00
ZDZ24	NNP	100.00	0.59	0.01
SYG01	NNP	100.00	0.71	0.01
SYG02	NNP	99.84	0.37	0.07
SYG03	NNP	99.91	0.01	0.00
SYG04	NNP	100.00	0.85	0.01
SYG05	NNP	100.00	0.19	0.00
SYG06	NNP	100.00	0.43	0.00
SYG07	NNP	100.00	0.56	0.00
SYG08	NNP	100.00	0.01	0.00
SYG09	NNP	100.00	0.21	0.00
SYG10	NNP	100.00	0.98	0.01
SYG11	NNP	100.00	0.17	0.00

SYG12	NNP	100.00	0.68	0.00
SYG13	NNP	100.00	0.99	0.01
SYG14	NNP	100.00	0.06	0.00
SYG15	NNP	100.00	0.20	0.00
SYG16	NNP	99.23	0.20	0.10
SYG17	NNP	99.98	0.15	0.02
SYG18	NNP	100.00	0.18	0.00
SYG19	NNP	100.00	0.74	0.00
SYG20	NNP	100.00	0.94	0.00
SYG21	NNP	100.00	0.09	0.00
SYG22	NNP	100.00	0.83	0.01
SYG23	NNP	100.00	0.29	0.00
SYG24	NNP	100.00	0.07	0.00
DFG01	SNP	98.91	0.04	0.23
DFG02	SNP	100.00	0.00	0.38
DFG03	SNP	100.00	0.00	0.33
DFG04	SNP	100.00	0.00	0.79
DFG05	NNP	99.91	0.60	0.12
DFG06	SNP	100.00	0.00	0.13
DFG07	SNP	100.00	0.00	0.35
DFG08	SNP	99.98	0.00	0.06
DFG09	SNP	76.17	0.07	0.13
DFG10	SNP	100.00	0.00	0.36
DFG11	SNP	100.00	0.00	0.79
DFG12	SNP	100.00	0.00	0.11
DFG13	SNP	100.00	0.00	0.61
DFG14	SNP	100.00	0.00	0.64
DFG15	SNP	99.99	0.00	0.11
DFG16	SNP	100.00	0.00	0.76
DFG17	SNP	100.00	0.00	0.73
DFG18	SNP	100.00	0.00	0.25
DFG19	SNP	100.00	0.00	0.27
DFG20	SNP	100.00	0.00	0.19
DFG21	SNP	99.95	0.02	0.27
DFG22	SNP	100.00	0.00	0.22
DFG23	SNP	100.00	0.04	0.67
DFG24	SNP	100.00	0.00	0.14
YGD01	SNP	100.00	0.00	0.37
YGD02	SNP	99.99	0.00	0.19
YGD03	SNP	99.82	0.00	0.02
YGD04	SNP	100.00	0.00	0.40
YGD05	SNP	100.00	0.00	0.37
YGD06	SNP	100.00	0.00	0.26
YGD07	SNP	100.00	0.00	0.05

YGD08	SNP	99.50	0.07	0.34
YGD09	SNP	100.00	0.00	0.70
YGD10	SNP	100.00	0.00	0.91
YGD11	SNP	100.00	0.00	0.31
YGD12	SNP	100.00	0.00	0.50
YGD13	SNP	100.00	0.00	0.34
YGD14	SNP	100.00	0.00	0.33
YGD15	SNP	100.00	0.00	0.33
YGD16	SNP	99.99	0.00	0.06
YGD17	SNP	99.88	0.03	0.31
YGD18	SNP	100.00	0.00	0.86
YGD19	SNP	100.00	0.00	0.32
YGD20	SNP	100.00	0.00	0.42
YGD21	SNP	100.00	0.00	0.24
YGD22	SNP	100.00	0.00	0.00
YGD23	SNP	100.00	0.00	0.44
YGD24	SNP	100.00	0.00	0.35
LS01	SNP	100.00	0.00	0.51
LS02	SNP	100.00	0.00	0.24
LS03	SNP	100.00	0.00	0.11
LS04	SNP	100.00	0.00	0.02
LS05	SNP	100.00	0.00	0.06
LS06	SNP	100.00	0.00	0.15
LS07	SNP	99.64	0.02	0.26
LS08	SNP	99.99	0.06	0.62
LS09	SNP	100.00	0.00	0.20
LS10	SNP	100.00	0.00	0.21
LS11	SNP	100.00	0.00	0.79
LS12	SNP	100.00	0.00	0.35
LS13	SNP	100.00	0.03	0.74
LS14	SNP	99.99	0.00	0.14
LS15	SNP	92.80	0.03	0.10
LS16	SNP	100.00	0.00	0.42
LS17	SNP	100.00	0.00	0.21
LS18	SNP	100.00	0.10	0.82
LS19	SNP	99.99	0.00	0.06
LS20	SNP	99.99	0.00	0.00
LS21	SNP	100.00	0.00	0.55
LS22	SNP	99.82	0.00	0.01
LS23	SNP	100.00	0.00	0.01
LS24	SNP	100.00	0.02	0.69
JQD01	SNP	99.95	0.00	0.11
JQD02	SNP	100.00	0.00	0.85
JQD03	SNP	99.99	0.00	0.14

JQD04	SNP	100.00	0.00	0.27
JQD05	SNP	99.59	0.06	0.34
JQD06	SNP	100.00	0.03	0.67
JQD07	SNP	100.00	0.00	0.18
JQD08	SNP	99.15	0.11	0.39
JQD09	SNP	100.00	0.00	0.08
JQD10	SNP	100.00	0.01	0.60
JQD11	SNP	100.00	0.00	0.28
JQD12	SNP	100.00	0.00	0.00
JQD13	SNP	100.00	0.00	0.57
JQD14	SNP	99.97	0.01	0.26
JQD15	SNP	100.00	0.00	0.23
JQD16	SNP	99.98	0.00	0.06
JQD17	SNP	100.00	0.00	0.02
JQD18	SNP	100.00	0.00	0.08
JQD19	SNP	100.00	0.00	0.56
JQD20	SNP	100.00	0.00	0.55
JQD21	SNP	100.00	0.00	0.31
JQD22	SNP	100.00	0.00	0.35
JQD23	SNP	99.99	0.00	0.07
JQD24	SNP	100.00	0.01	0.67

■ Figures

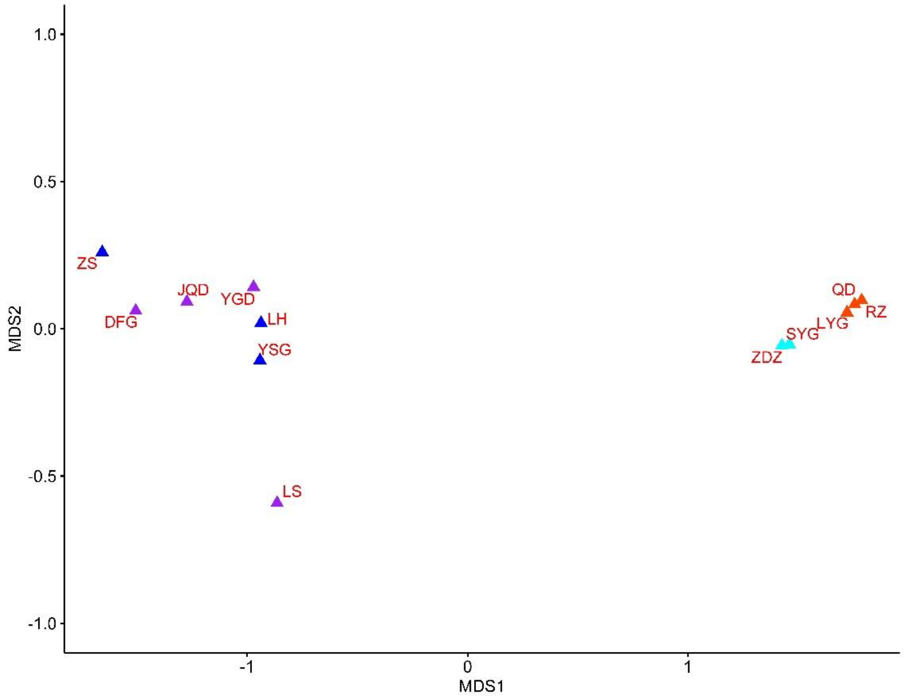

Fig.S1 Multidimensional scaling analyses (MDS) of *Φ*st based on mtDNA ND6 sequences for 12 populations.

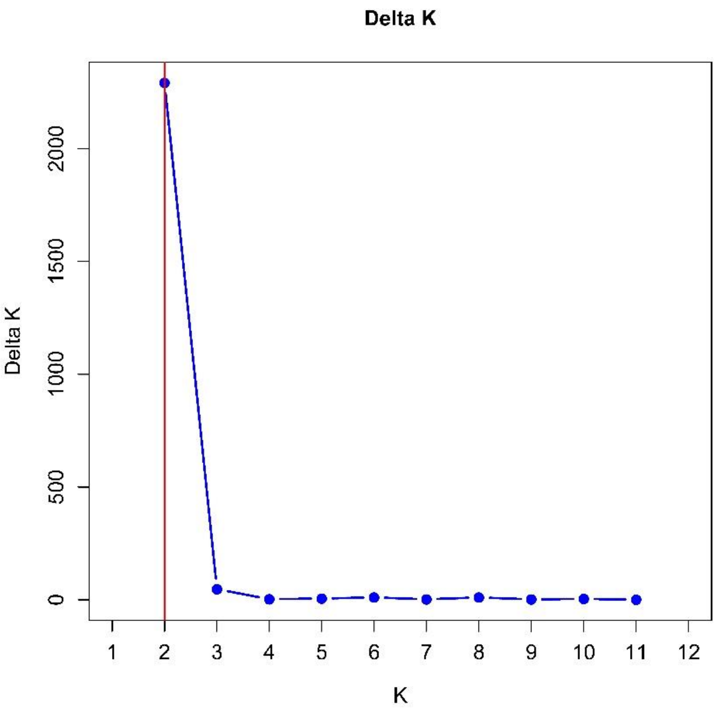

Fig. S2 Plot of Delta K values generated by StructureSelector for STRUCTURE runs based on 12 microsatellite loci.

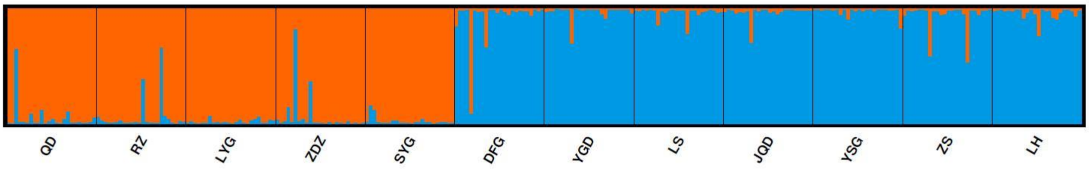

Fig. S3: STRUCTURE output with K = 2 showing population substructure among 12 populations based on eight microsatellite loci.

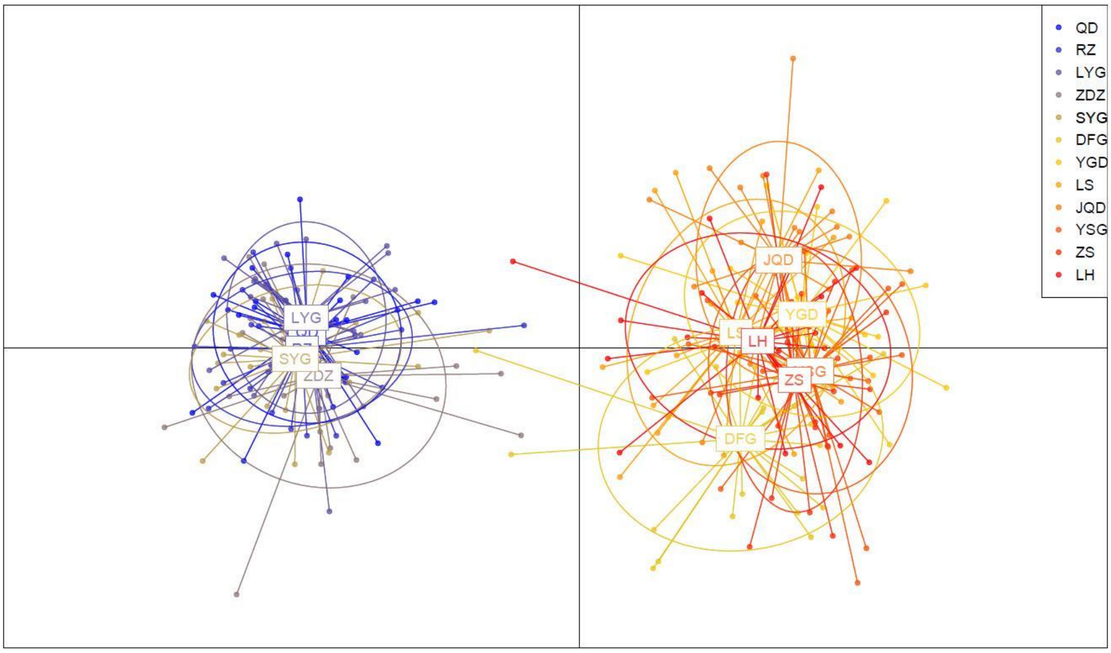

Fig. S4: Plot of DAPC scatter based on eight loci, generated using first two principal components of the 11 components, explaining 89.77% variance in total. Dots represent individuals from the twelve locations.
